# The 2011 eruption of Nabro volcano, Eritrea: perspectives on magmatic processes from melt inclusions

**DOI:** 10.1007/s00410-017-1425-2

**Published:** 2017-11-27

**Authors:** Amy Donovan, Jon Blundy, Clive Oppenheimer, Iris Buisman

**Affiliations:** 1 Department of Geography, King’s College London, London, UK; 2 School of Earth Sciences, University of Bristol, Bristol, BS8 1RJ UK; 3 Department of Geography, University of Cambridge, Cambridge, UK; 4 Department of Earth Sciences, University of Cambridge, Cambridge, UK

**Keywords:** Melt inclusions, Afar volcanism, Magmatic processes, Nabro volcano

## Abstract

The 2011 eruption of Nabro volcano, Eritrea, produced one of the largest volcanic sulphur inputs to the atmosphere since the 1991 eruption of Mt. Pinatubo, yet has received comparatively little scientific attention. Nabro forms part of an off-axis alignment, broadly perpendicular to the Afar Rift, and has a history of large-magnitude explosive silicic eruptions, as well as smaller more mafic ones. Here, we present and analyse extensive petrological data obtained from samples of trachybasaltic tephra erupted during the 2011 eruption to assess the pre-eruptive magma storage system and explain the large sulphur emission. We show that the eruption involved two texturally distinct batches of magma, one of which was more primitive and richer in sulphur than the other, which was higher in water (up to 2.5 wt%). Modelling of the degassing and crystallisation histories demonstrates that the more primitive magma rose rapidly from depth and experienced degassing crystallisation, while the other experienced isobaric cooling in the crust at around 5 km depth. Interaction between the two batches occurred shortly before the eruption. The eruption itself was likely triggered by recharge-induced destabilisation of vertically extensive mush zone under the volcano. This could potentially account for the large volume of sulphur released. Some of the melt inclusions are volatile undersaturated, and suggest that the original water content of the magma was around 1.3 wt%, which is relatively high for an intraplate setting, but consistent with seismic studies of the Afar plume. This eruption was smaller than some geological eruptions at Nabro, but provides important insights into the plumbing systems and dynamics of off-axis volcanoes in Afar.

**Electronic supplementary material:**

The online version of this article (10.1007/s00410-017-1425-2) contains supplementary material, which is available to authorized users.

## Introduction

**Fig. 1:**
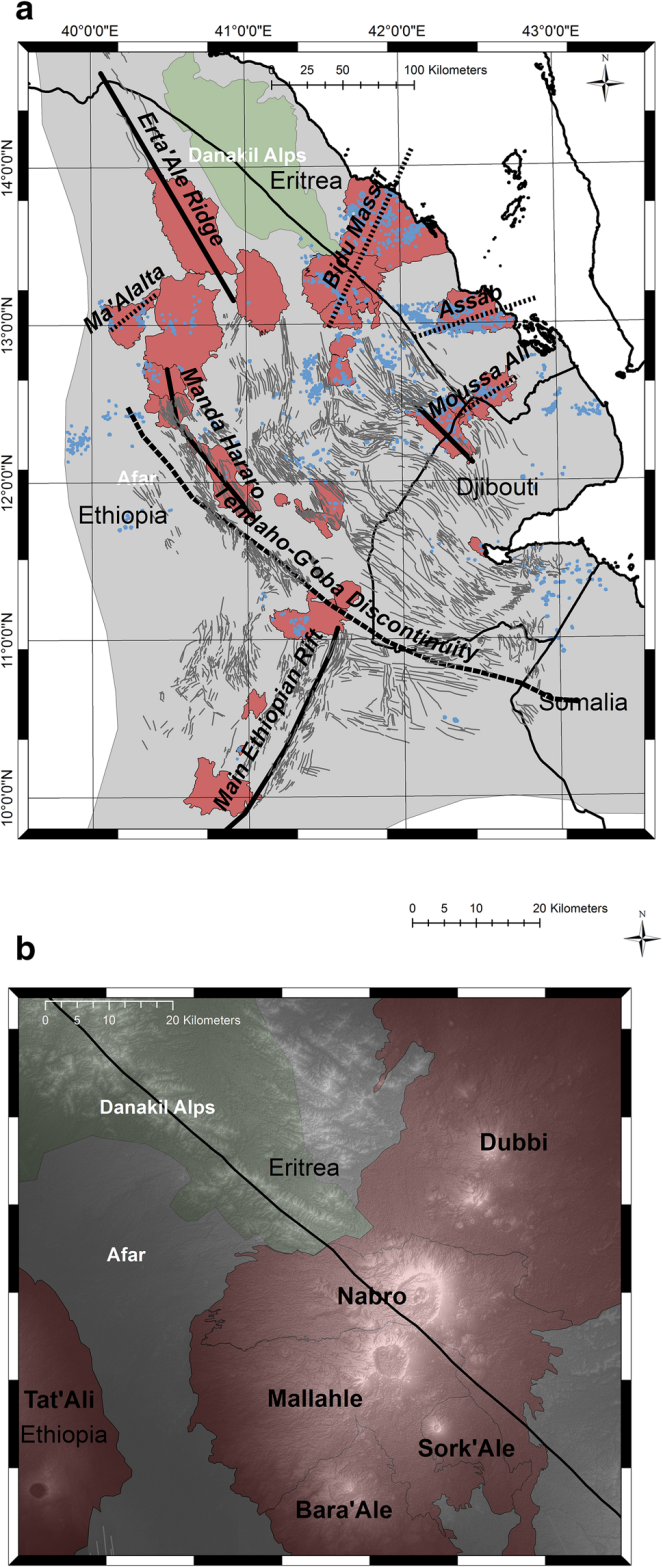
**a** Map of the Afar Depression, showing the tectonic structures and main volcanic fields discussed in this paper. Redrawn from Barberi and Varet (1977), using Landsat 8 imagery. Dark red areas are the main volcanic fields. Thin black lines are faults and linear structures; thick black lines are spreading centres; short-dashed lines are the marginal ranges after Barberi et al. 1974a; and the long-dashed line is the Tendaho-G’oba Discontinuity. The Danakil Alps (part of the Danakil Horst) are shown in light green. Small blue dots are spatter cones. Note the NW–SE arrangement of spatter cones to the south of Mallahle, perpendicular to the Bidu alignment. **b** Close-up of Nabro volcano (colour scheme as for **a**), using a digital elevation model (ASTER GDEM generated by METI and NASA) to highlight the calderas

Nabro volcano in the northern Afar Depression, Eritrea, is a stratovolcano with a breached caldera opening to the south-west. It is the highest topographical feature in the region, and abuts the caldera of Mallahle volcano at the international border with Ethiopia (Fig. 1), forming part of an alignment known as the Bidu Massif. The volcano was little heard of until 2011 when it was the site of a substantial explosive and effusive eruption—the first in recorded history—that precipitated a significant humanitarian crisis, disrupted aviation and yielded one of the largest sulphur inputs to the atmosphere since the 1991 eruption of Mount Pinatubo (Goitom et al. 2015; Theys et al. 2013). Here, we present the first detailed petrological and geochemical study of the eruptive products, focusing on the tephra deposits. Our aims are to characterise the magma(s) involved in the eruption, evaluate trigger mechanisms, define degassing mechanisms and trajectories, and understand the high sulphur yield.

### Regional background

The Afar Depression is an active spreading zone at the boundary between the Arabian and Nubian plates, lying just to the west of the triple junction with the Somalian plate (Fig. 1). Eruption of the flood basalts of the Ethiopian Traps circa 30 Ma ago (Courtillot et al. 1999; Schilling 1973) presaged the separation of Arabia and Africa and formation of the Afar Rift (Furman et al. 2006; Hofmann et al. 1997). Most lavas now exposed in the Afar Depression are much younger than the Traps and record the transition to ocean-floor magmatism over the past 10 Ma (Barrat et al. 2003; Hagos et al. 2016).

Despite the logistical challenges of carrying out fieldwork in Afar, several decades of research underpin our current understanding of the regional volcanism and rifting (Ayele et al. 2007, 2009; Barberi et al. 1974a, b; Ferguson et al. 2010; Field et al. 2012a, b, 2013; Hammond et al. 2013, 2011; Keir et al. 2013, 2011; Lahitte et al. 2003). Expeditions in the 1970s documented Afar volcanism at reconnaissance scale (Barberi et al. 1974a, b, 1970; Civetta et al. 1975). Barberi et al. (1970) distinguished between the axial ranges (Erta’Ale and Alayta) and marginal ranges exemplified by Ma’Alalta and Dabbahu (Field et al. 2012b, 2013; Wiart and Oppenheimer 2005). The latter are generally more alkaline and show signs of crustal contamination (Civetta et al. 1975; De Fino et al. 1978; Ottonello et al. 1978). Barberi et al. (1974a) went on to describe the marginal ranges as “transverse” ranges, drawing an analogy with those that offset the Mid Atlantic Ridge, an idea that has not been universally accepted. De Fino et al. (1978), for example, attributed the difference in composition between the marginal and axial ranges to differing redox conditions, possibly controlled by mantle temperature variations. Conversely, Civetta et al. (1975) argued for structural control on the depth of mantle melting.

More recent studies of the Afar region and Ethiopian Rift Valley have revealed physical and chemical heterogeneity [e.g., (Ebinger et al. 2010; Ebinger and Sleep 1998; Furman et al. 2006; Keir et al. 2011; Pik et al. 2008, 2006) Rooney et al. 2012a]; Hammond et al. (2011) showed that there is a considerable variation in Moho depth across Afar, even within the central rift area. Their study area does not quite reach the Bidu Massif, but points to a crust thickness of about 20 km in the region of the Danakil Horst (Fig. 1). Hammond et al. (2013) inferred from seismic data that several diapiric mantle upwellings are present under off-axis volcanoes, including Nabro. They suggested that these might arise from elevated H_2_O contents and enhanced melting along the edges of thicker continental lithosphere. Volcanism across the Red Sea in Yemen, likely related to the Afar melting anomaly [e.g., (Baker et al. 1998; Ukstins et al. 2002)] shows evidence of elevated H_2_O content (Baker et al. 1998) that might lower the solidus sufficiently to enhance melt production. Elevated mantle temperatures have also been invoked as the cause of voluminous mantle melting in the region (Rooney et al. 2012b).

Melt inclusion studies from the region are sparse. Field et al. (2012a) studied melt inclusions in basaltic lavas from Erta ‘Ale volcano, erupted in 2010. These contained low H_2_O contents (< 0.13 wt% H_2_O), thought to reflect extensive prior degassing of the magma at shallow depth rather than initial low magmatic H_2_O per se. Melt inclusion data for silicic lavas from Dabbahu volcano indicated a shallow magma reservoir, with crystallisation driven by cooling rather than decompression (Field et al. 2012b). More broadly, basaltic melt inclusion studies of intraplate and rift-related volcanic systems have focussed primarily on Hawaii [e.g., (Edmonds et al. 2013; Norman et al. 2002; Sides et al. 2014)], Iceland (Maclennan 2008; Neave et al. 2014; Winpenny and Maclennan 2011) and Piton de la Fournaise (Bureau et al. 1998) in addition to studies of Mid-Ocean Ridge Basalts [MORB; e.g., (Font et al. 2007; Nielsen et al. 1995)]. These studies reveal that there is considerable variation in mantle volatile content and provide evidence that mantle plumes may be enriched in volatiles compared to MORB (Dixon and Clague 2001; Dixon et al. 2002; Wallace 1998). Detailed studies of Icelandic magmas show a complex picture of melt evolution, mixing, and assimilation in the crust [e.g., (Shorttle et al. 2013; Winpenny and Maclennan 2011)], and this points to both the heterogeneity of the mantle source and the varied evolutionary paths that a single magma may take within the crust. The Afar region bears comparison with Iceland, in that it is a subaerial spreading centre influenced by at least one mantle plume (George et al. 1998; Hofmann et al. 1997; Pik et al. 2006; Schilling 1973).

### Eruptive history of Nabro

The eruptive history of the Bidu massif is poorly known. There is evidence of large-magnitude caldera-forming eruptions at Nabro and Mallahle at the south-western end of the Bidu Massif, where rock compositions range from primitive basalt to trachyte and rhyolite (Wiart and Oppenheimer 2005). Nabro and Mallahle are two of several volcanic centres lying along the NE–SW-oriented Bidu alignment; the others include Dubbi volcano to the northeast, and Kod Ali in the Red Sea (Fig. 1). The Edd volcanic field, which lies to the north of Nabro and Dubbi, comprises lavas erupted from Dubbi volcano and associated fissures (De Fino et al. 1978). To the ENE of Dubbi, Kod Ali volcano sits offshore on a small island; its eruptive products contain abundant ultramafic nodules (Hutchison and Gass 1971). When Nabro erupted in 2011, the eruption was widely thought to be from Dubbi until satellite imagery became available (Theys et al. 2013).

There has been very little previous work on the petrology of Nabro volcano, the only study being the PhD thesis of Wiart (2005). This focussed on Dubbi, but includes whole-rock analyses of ten samples from Nabro, all of which are silicic (63–74 wt% SiO_2_), while noting the presence of more mafic units, interpreted as recent. Wiart and Oppenheimer (2005), using satellite data, described three large ignimbrite deposits, which they termed the Western, the Eastern, and the South Eastern Ignimbrites. Wiart and Oppenheimer (2005) attributed the Western Ignimbrite to Nabro and the other two to Mallahle, suggesting that all three ignimbrites may have formed in a single, very large event. In addition to the ignimbrites, they identify at least two different effusive trachytic eruptions, and a number of rhyolite flows, obsidian flows, and the recent basalts. Wiart and Oppenheimer (2005) further compared the Nabro-Mallahle volcanic system with volcanism on the other side of Afar, at Ma’alalta volcano (Fig. 1). In estimating the volume of these marginal units on the flanks of the Afar Depression, they noted that volumetrically, they are comparable with eruptive units in the rift axis itself—illustrative of voluminous off-axis volcanism.

**Fig. 2:**
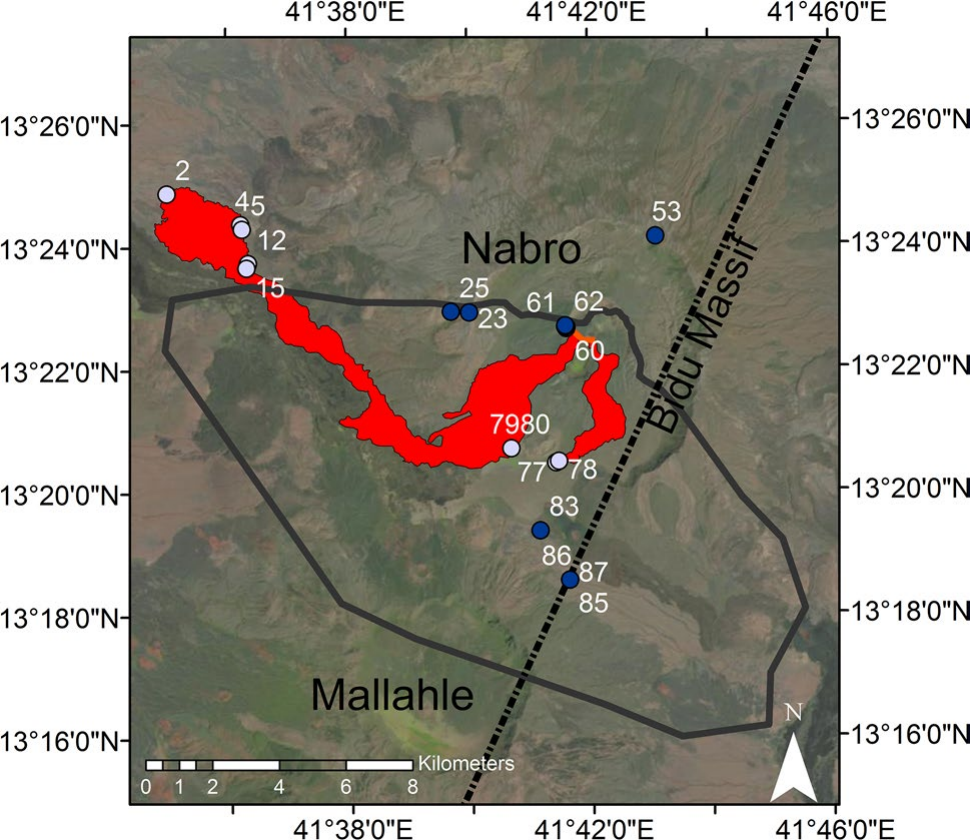
Landsat image of Nabro and Mallahle volcanoes (acquired before the 2011 eruption), with a sketch of the deposits from the 2011 eruption based on ALI imagery. Lava flows are shown in red. Black outline denotes the main 2011 tephra field, which was sampled in Eritrea only. Sample locations are shown as numbered blue circles. Light blue denotes lava samples; dark blue denotes tephra samples (the focus of this paper). Dotted line indicates the axis of the Bidu Massif

During the 2011 eruption, activity was concentrated along a rift at 90° to the Bidu Massif (Goitom et al. 2015). Two major lava flows were produced, one of which extended ~ 15 km to the northwest of the volcano; the other was shorter and moved southwards within the caldera (Fig. 2; Goitom et al. 2015). The tephra field was well-defined to the south-west of the edifice (towards and blanketing Mallahle volcano). During the eruption, seismicity was also detected under Mallahle, raising the possibility of an underground connection between the two volcanoes [e.g., Hamlyn et al. (2014)]. The 2011 eruption represents an unprecedented opportunity to study an eruption at one of the large caldera volcanoes on the margins of the Afar Depression. Here, we analyse melt inclusions and minerals from the 2011 tephra to investigate the history of the magma that fed the eruption. Our key objectives are to constrain the volatile budget and pre-eruptive storage conditions of the 2011 magma and to identify potential eruption triggers. More broadly, these objectives allow us to contribute to understanding off-axis volcanism in Afar.

## Methods

Fresh tephra samples (size < 2 cm^3^) were crushed using a jaw crusher and ball mill. Whole-rock analyses were made using X-ray fluorescence spectroscopy at Leicester University (major elements). Trace elements were measured by Inductively Coupled Plasma Mass Spectrometry (ICPMS) using a Perkin Elmer Elan DRC II instrument at the University of Cambridge, following dissolution in nitric acid and hydrofluoric acid. Phenocrysts of olivine and plagioclase were extracted from crushed samples and mounted in epoxy resin, ground and polished, and then imaged using a Hitachi S-3500N scanning electron microscope at the University of Bristol. Variable vacuum mode was used to avoid the need to carbon-coat the samples before they were ion-probed. Melt inclusions were identified and screened for devitrification textures. Most of the melt inclusions in olivine were partially devitrified; hence, most inclusions studied here are plagioclase hosted; all were glassy with no evident cracks or breaches. Most lacked shrinkage bubbles; those that did have them were noted. Melt inclusions were not rehomogenised prior to analysis for several reasons: heating experiments can affect the oxidation state of the inclusion, promoting diffusion of H, for example (Rowe et al. 2007); it is easy to over- or under-heat melt inclusions (Nielsen 2011); and heating may cause physical changes such as cracks (Nielsen et al. 1995). The potential role of natural post-entrapment processes is discussed in “Melt inclusion compositions”.

Polished grain mounts were gold-coated; 130 melt inclusions and four matrix glasses were analysed for volatiles and trace elements at the NERC Ion Microprobe Facility in Edinburgh. CO_2_ was measured as ^12^C^+^ on the Cameca ims-1270 instrument; H_2_O (as ^1^H^+^) and “light” (< 47 amu) trace elements were measured on the Cameca ims-4f. Some inclusions were selected for additional “heavy” (> 30 amu) trace element measurement on the ims-4f. For each inclusion, analyses were made in the order: CO_2_, H_2_O and light traces, and (where selected) heavy traces, in three separate measurements. Titanium content was used to check for consistency across all sets, and with the electron probe data.

Analyses of CO_2_ on the Cameca ims-1270 used a 3.8 nA primary beam of negatively charged oxygen dimer ions (O_2_^−^) at 22 keV net impact energy focussed to a spot of approximately 15–20 µm. A 1-min pre-sputter with a 10 µm^2^ raster was applied. Twenty cycles were recorded and the final eight retained. Secondary ions were collected at 10 kV with a 50 eV offset, at sufficient mass resolution to fully resolve ^24^Mg^2+^ from ^12^C^+^. The CO_2_ concentrations were defined by a working curve using four basaltic glass standards (Lesne et al. 2011b), with a range in composition of 900–2550 ppm CO_2_ and a volatile-free glass blank. Backgrounds were recorded throughout the measurements using the olivine and plagioclase host crystals. Standards were measured at the start and end of each session, and data and working curves are provided as supplementary data.

Analyses for water and light trace elements on the Cameca ims-4f used a 15 µm 5–6 nA primary beam of O^−^ ions with 15 keV net impact energy. A 1-min pre-sputter of 10 µm^2^ was applied. Secondary ions were collected at 4.5 kV with a 75 eV offset. A field aperture was employed to restrict secondary ion collection to the central 8–10 µm of the sputtered pit to reduce background. Heavy trace elements were also measured on the Cameca ims-4f using a 15 µm 5–6 nA beam with a 75 eV offset to reduce molecular ion transmission. Positive secondary ions were measured from the full 25 µm image field. Analyte isotopes were normalised to ^30^Si and then corrected for SiO_2_ concentration based on EPMA measurements. Interferences from the light REE oxides and BaO on the heavy REE were removed by peak-stripping. Backgrounds were recorded throughout the water measurements using the olivine and plagioclase host crystals. Standards were measured at the start and end of each session. The fully propagated Gaussian errors are displayed on the data tables for H_2_O and CO_2_. For the trace elements, errors are ± 10%. Following ion-probe analysis, major and minor element (including S, Cl, and F) analysis was undertaken at the University of Cambridge using a Cameca SX100 electron microprobe. Inclusions and host glasses were measured with a 15 kV accelerating voltage, 6 nA beam current, and a defocused 10 µm beam. Na was always analysed first to minimise alkali loss. Minerals were analysed both in the proximity of each melt inclusion and at their rims, using 15 kV, 20 nA focussed 1 µm beam. Major elements were counted for 30–60 s and volatile elements for 90–120 s. Particular care was taken to measure *F* to avoid *F* migration in the glass. In addition to the inclusions and their hosts, measurements of phenocryst and groundmass minerals were made on thin sections of both lavas and tephra from several different samples.

A number of statistical tests were performed on the data set. Where data were grouped—for example, by rock sample—the Kruskal–Wallis test was applied to look for differences between groups. Where there were only two groups, the Kolmogorov–Smirnov test was used. Both these tests are appropriate for non-parametric data sets and care was taken to ensure that small groups did not unduly influence the result. For data that had been summed to 100% and where a transformation was indicated by the distribution, the data were first transformed using a square root transformation for small values and an arcsin transformation for larger values (i.e., SiO_2_).

## Results

### Whole-rock and glass compositions

**Fig. 3:**
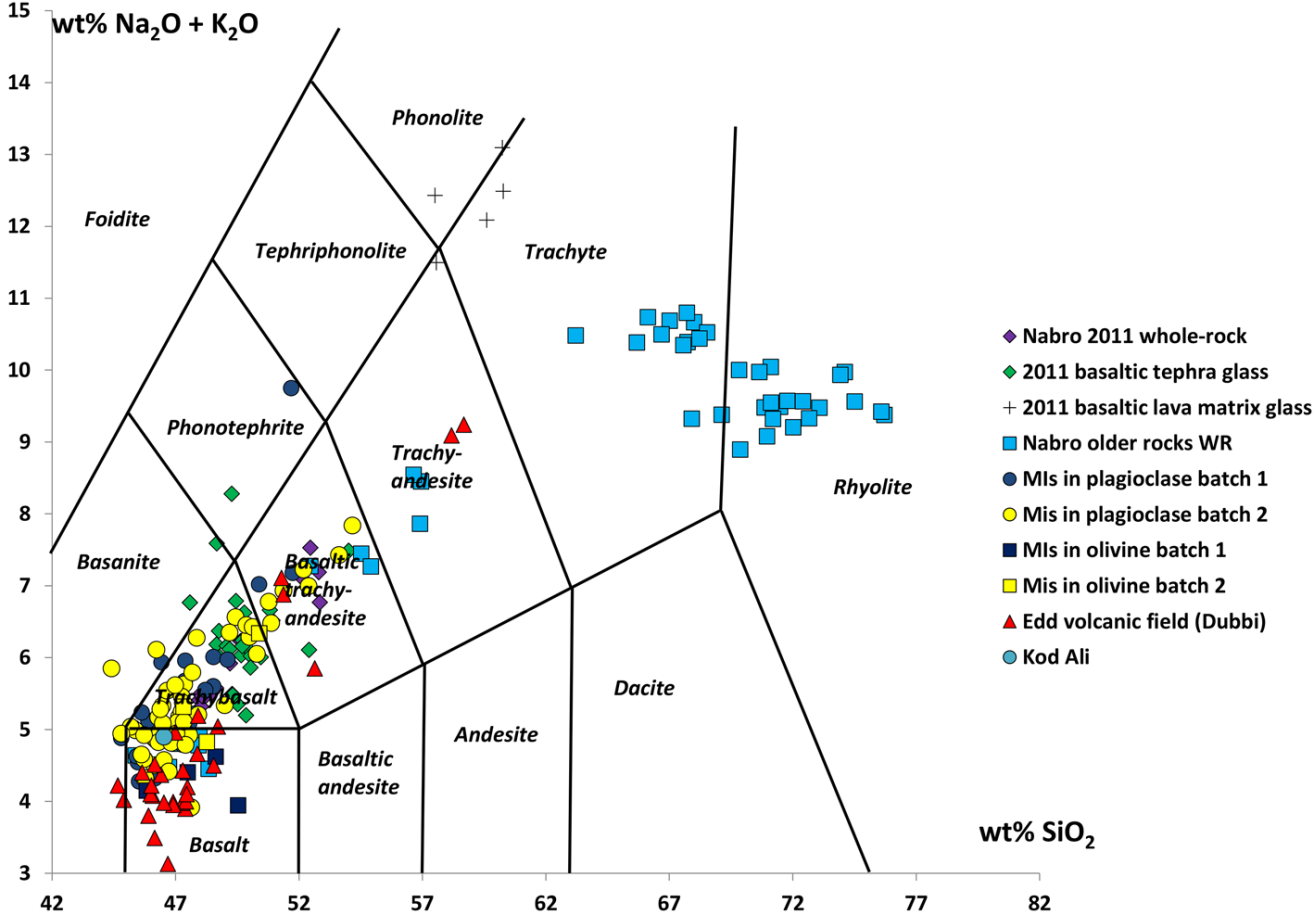
Total alkali–silica diagram after LeBas et al. (1986) showing the whole rock (WR), glass, and melt inclusion compositions from the 2011 eruption of Nabro. Also shown are older rocks from Nabro and samples from Edd volcanic field (De Fino et al. 1978) and a single sample from Kod Ali (an island in the Red Sea situated on the Bidu lineament; Hutchison and Gass 1971). All data are normalised to 100% anhydrous. The two batches of the 2011 magma are explained in “Petrography and mineralogy of 2011 samples”. Note abscissa on axes

A total alkali–silica diagram is shown in Fig. 3. The compositions of the 2011 whole rocks (Table 1) and melt inclusions are broadly similar to older mafic rocks found on Nabro and lie on an apparent fractionation trend that extends to the rhyolitic compositions of the ancient ignimbrites. However, lava matrix glasses from the 2011 eruption are displaced to higher total alkalis, reaching eventually the phonolite field (Fig. 3). This is particularly the case for the lava flows, where plagioclase and clinopyroxene crystallisation dominate. Representative whole-rock compositions are shown in Table 1.

**Table 1:** Representative whole-rock compositions

	NAB-11-12	NAB-11-2	NAB-11-4
	Tephra	Lava	Lava
SiO_2_	48.63	49.63	52.10
TiO_2_	3.11	2.86	2.40
Al_2_O_3_	15.61	15.77	16.24
Fe_2_O_3_	12.17	11.83	10.73
MnO	0.21	0.22	0.23
MgO	4.79	4.26	3.35
CaO	8.63	7.79	6.85
Na_2_O	4.17	4.57	5.21
K_2_O	1.41	1.66	1.90
P_2_O_5_	1.02	0.98	0.90
SO_3_	0.06	0.03	0.02
Mg#	41.24	39.10	35.76
LOI	− 0.49	− 0.55	− 0.51
Total	99.33	99.07	99.42
Li	9.64	9.54	11.53
Be	1.96	2.07	2.30
Sc	41.14	26.76	22.93
V	255.51	191.52	127.56
Cr	51.72	32.48	10.35
Co	28.83	22.75	14.67
Ni	28.29	21.60	6.76
Cu	33.60	27.34	15.62
Zn	123.73	128.19	130.52
Ga	24.01	24.67	25.41
Rb	37.25	42.49	50.12
Sr	783.87	759.62	772.04
Y	44.68	48.79	52.29
Zr	303.21	352.95	405.57
Nb	77.05	86.86	97.49
Sn	2.23	2.77	2.60
Cs	0.30	0.33	0.41
Ba	451.85	518.98	608.96
La	56.38	64.99	71.89
Ce	120.13	134.48	148.07
Pr	14.75	16.51	18.22
Nd	62.73	67.67	73.17
Sm	12.22	13.24	13.81
Eu	4.09	4.43	4.79
Gd	10.52	11.31	11.80
Tb	1.58	1.67	1.80
Dy	8.57	9.20	9.86
Ho	1.58	1.72	1.90
Er	4.08	4.45	4.81
Tm	0.57	0.61	0.67
Yb	3.28	3.61	4.02
Lu	0.47	0.51	0.58
Hf	7.37	8.28	9.38
Ta	4.82	5.39	5.94
Tl	0.04	0.04	0.02
Pb	3.25	3.29	3.85
Th	5.65	6.61	7.62
U	1.53	1.79	2.09

Major elements measured by XRF, trace elements by ICPMS. All data plotted in this paper are available in Supplementary Data

### Petrography and mineralogy of 2011 samples

The 2011 samples contain between 15 and 30 vol% phenocrysts. Lava samples are highly crystalline (< 3% interstitial glass) and contain abundant groundmass plagioclase (~ 70%), with appreciable clinopyroxene (~ 20%) and less than 10% olivine and Fe–Ti oxides. Lavas are not considered further in this paper as all studied melt inclusions are from rapidly-cooled, small tephra clasts.

The 2011 tephras contain phenocrysts of olivine, clinopyroxene, plagioclase, and Fe–Ti oxides, with minor apatite. Olivines (Fo_80−85_) are typically euhedral with narrow, more fayalite-rich rims (Fo_70−75_). However, some olivines are reverse zoned (Fo_60_ in the core to Fo_70_ in the rim) or unzoned (Fig. 6). Olivine phenocrysts up to 1000 µm in length contain inclusions of chromite, sometimes with magnetite mantles. Chromites also occur on the edges of olivines, always with a magnetite rim. Clinopyroxene (up to 1000 µm in length) is present in two main populations—reverse zoned (~ Mg#^1^ 50–60 in the core, ~ Mg# 75–80 at rim) and unzoned (Mg# 75–80). Plagioclase is normally zoned, with cores up to ~ An_80_ and rims varying from An_50_ to An_75_. The largest crystals are > 2000 µm in length. Both ilmenite and magnetite are present (typically 200 µm, but some larger), as are large fluorapatite crystals (> 500 µm).

**Fig. 4:**
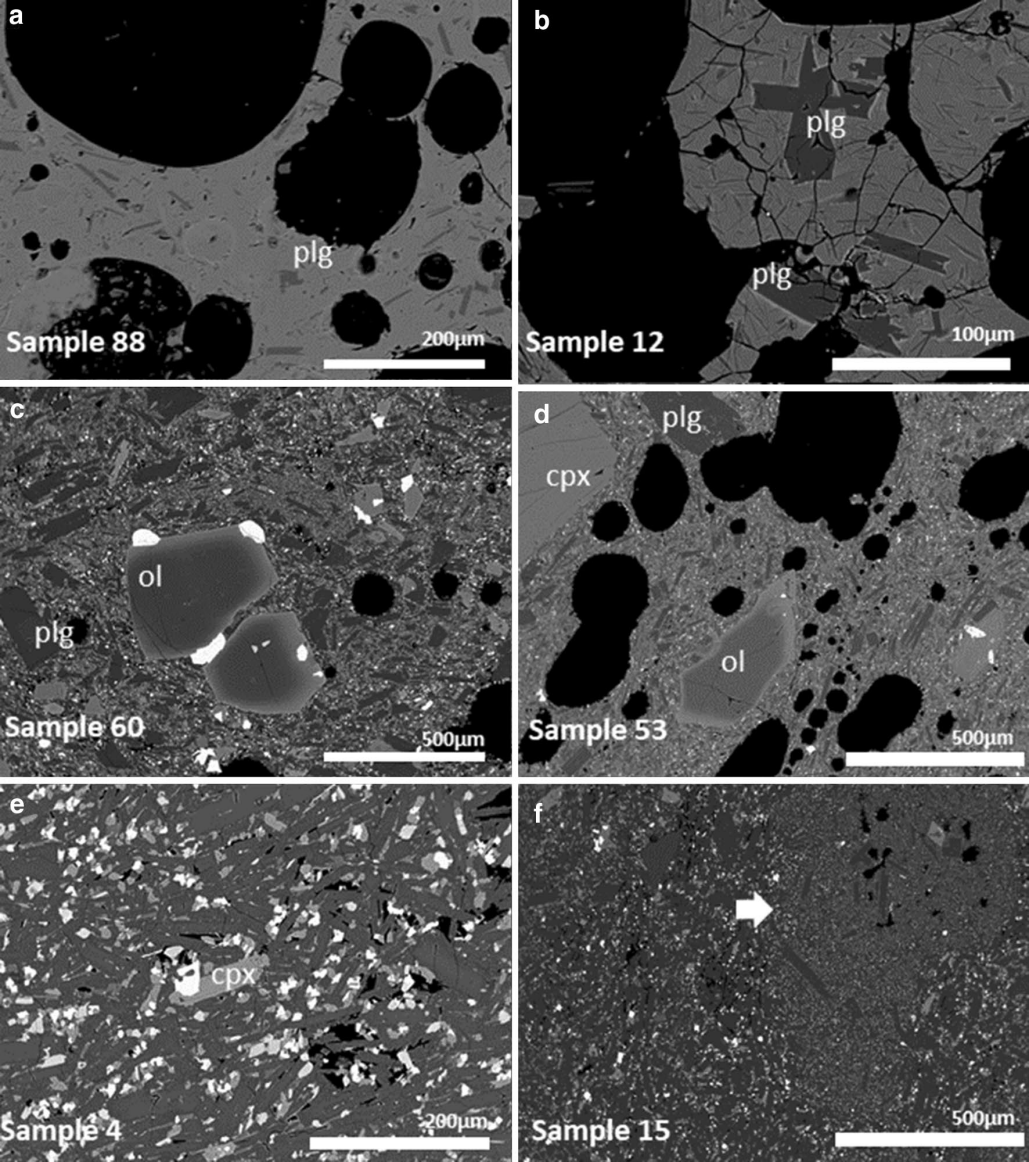
Back-scattered electron micrographs of the 2011 eruptive products. **a, b** Show tephra “Batch 1”, **c, d** show tephra “Batch 2”, and **e, f** are from lava samples. Note the presence of a vesicular, finer grained component in **f** (arrow)—this phenomenon is also found in the tephra

There are two dominant primary textures in the tephra (sometimes found mingled), as well as a further fine-grained texture that is found mingled in lavas and attributed to mingling between the two tephra textures with additional cooling (see, for example, Fig. 4f). We identify two distinct batches of magma based on the primary textural features of tephra groundmass (Table 2). Batch 1 is very glassy and almost all of the phenocrysts and microlites are plagioclase, with lesser clinopyroxene; phenocrysts are often unzoned (Fig. 4a, b). Batch 2 is highly crystalline (> 70% total crystals) and dominated by plagioclase, followed by clinopyroxene, olivine, and oxides (Fig. 4c, d). Phenocrysts of these minerals and minor apatite occur, many showing zonation. While there are some compositional differences between the two batches of magma, bulk-rock and mineral chemistries are very similar. The whole-rock composition of the plagioclase-rich magma (Batch 1) is slightly richer in Al_2_O_3_ and MgO, and poorer in P_2_O_5_, compared with Batch 2.

**Table 2:** Average modal proportions of minerals and glass in the groundmass of the two main tephra textures (Batch 1 and Batch 2) and the proportion of phenocrysts relative to groundmass

Batch	%Plag	%Ol	%cpx	%Fe–Ti	%Glass	%Phenocrysts
1	24	2	2	0	72	29
2	50	7	15	8	20	15

Standard deviation is 10% for both types—there is considerable variation. All data obtained by point counting of thin sections (1000 points per section) and analysis of back-scattered electron micrographs in ImageJ

Also present in the rocks are larger crystals (> 1 mm), sometimes aggregated, which show disequilibrium textures and which we consider to be xenocrysts. These include plagioclase, olivine, clinopyroxene, orthopyroxene, and magnetite. One magnetite crystal (> 2 mm) is unzoned. Olivines (up to 2 mm in size) are more Mg-rich than Fo_85_, and clinopyroxene has Mg# ~ 80. Both show minor zoning towards Fe-rich compositions at their rims. One highly reacted anorthoclase crystal was also observed. Plagioclase megacrysts (> 2 mm) with high-An cores (> 85) occur rarely.

**Fig. 5:**
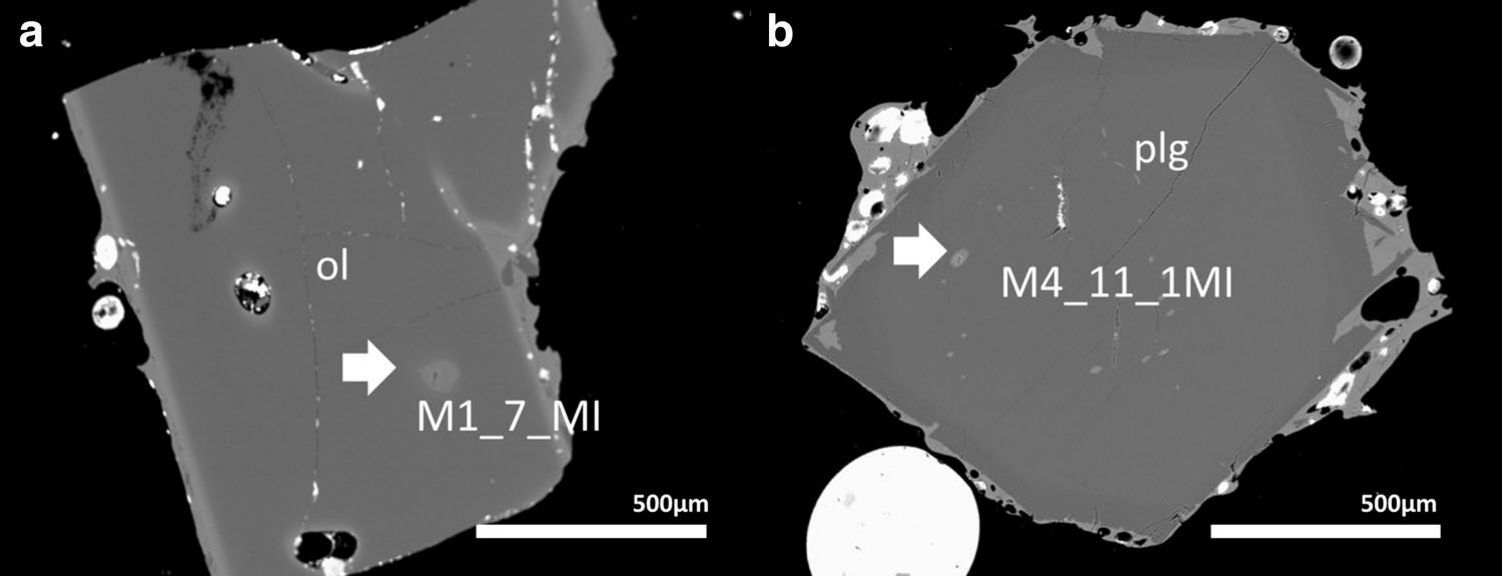
Examples of melt inclusions in olivine and plagioclase phenocrysts

**Fig. 6:**
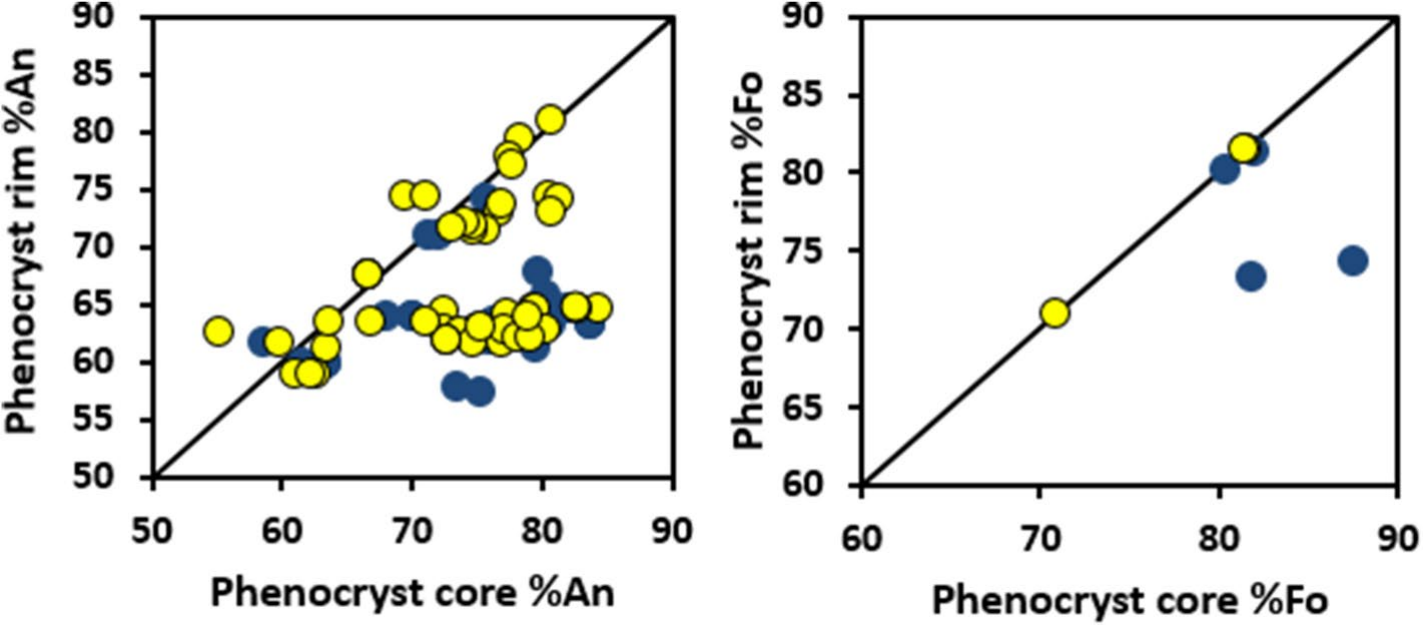
Rim and core compositions of the phenocrysts of plagioclase and olivine that hosted the melt inclusions. Batch 1 is blue and Batch 2 is yellow

The glassy melt inclusions studied here were mostly found in plagioclase phenocrysts, with a lesser number hosted by olivine (Fig. 5; Table 3). A few small melt inclusions in clinopyroxene were measured with the electron probe only. The host crystals generally follow the patterns above. In the plagioclase hosts, there are two sub groups, one with An_80−71_ and one with An_66−58_. Figure 6 shows the core and rim compositions of olivine and plagioclase crystals, whose melt inclusions we investigate here. For plagioclase hosts, in particular, there are two primary groups—normally zoned and unzoned (Fig. 6). There is no difference in host mineral compositions between the two texturally-identified magma batches.

**Table 3:** Representative melt inclusion host phenocryst compositions

a. Olivine	NAB11-S4-2-1	NAB11-M4-4-1	NAB11-M3-16-2
*Ol cores*			
SiO_2_	38.49	39.66	39.32
TiO_2_	0.02	0.02	0.03
Al_2_O_3_	0.04	0.06	0.05
FeOT	16.92	17.19	17.54
MnO	0.20	0.28	0.22
MgO	43.10	43.33	42.99
CaO	0.25	0.27	0.24
Na_2_O	0.01	0.02	
K_2_O	0.01	0.01	
P_2_O_5_	0.01	0.01	0.03
Cr_2_O_3_	0.04	0.01	0.00
NiO	0.23	0.25	0.19
Total	99.32	101.11	100.60
Fo	81.95	81.79	81.38
Fa	18.05	18.21	18.62
*Ol rims*			
SiO_2_	37.54	38.47	39.65
TiO_2_	0.02	0.05	0.03
Al_2_O_3_	0.04	0.03	0.04
FeOT	16.81	24.63	17.41
MnO	0.22	0.38	0.20
MgO	41.15	37.90	43.14
CaO	0.26	0.26	0.25
Na_2_O	0.03	0.03	
K_2_O	0.00	0.00	
P_2_O_5_	0.01	0.06	0.00
Cr_2_O_3_	0.02	0.02	0.01
NiO	0.20	0.11	0.19
Total	96.30	101.95	100.91
Fo	81.35	73.28	81.54
Fa	18.65	26.72	18.46

b. Plagioclase	NAB11-M4-11-1	NAB11-S4-16-2	NAB11-M4-6-2	NAB11-S4-11-1	NAB11-M4-22-1	NAB11-M4-18-2	NAB11-M1-3-1	NAB11-S4-19-1
*Plag cores*								
SiO_2_	46.95	48.47	50.22	46.95	52.99	48.58	52.42	51.09
TiO_2_	0.03	0.06	0.07	0.10	0.05	0.06	0.11	0.10
Al_2_O_3_	32.75	31.32	31.81	31.85	28.29	31.09	29.55	29.40
FeOT	0.52	0.59	0.71	0.58	0.49	0.58	0.45	0.48
MnO							0.03	
MgO	0.08	0.08	0.08	0.08	0.07	0.08	0.08	0.06
CaO	16.21	14.87	14.82	15.92	11.18	14.84	12.14	12.74
Na_2_O	1.91	2.78	3.08	2.36	4.88	2.84	4.36	3.94
K_2_O	0.10	0.12	0.15	0.11	0.20	0.13	0.19	0.18
SrO	0.16	0.22	0.17	0.16	0.21	0.11	0.15	0.15
Total	98.75	98.56	101.11	98.11	98.39	98.36	99.52	98.17
An	81.93	74.19	72.03	78.34	55.21	73.69	59.93	63.43
*Plag rims*								
SiO_2_	50.34	48.69	50.04	47.82	51.38	51.32	52.13	51.93
TiO_2_	0.17	0.08	0.07	0.06	0.12	0.09	0.12	0.11
Al_2_O_3_	29.65	30.68	31.44	32.70	29.38	29.69	29.25	29.17
FeOT	0.64	0.60	0.61	0.56	0.50	0.60	0.66	0.68
MnO							0.05	
MgO	0.09	0.09	0.07	0.08	0.11	0.09	0.08	0.11
CaO	12.86	14.27	14.38	16.03	12.65	12.65	12.49	12.34
Na_2_O	3.77	3.01	3.15	2.22	4.06	4.05	4.15	4.20
K_2_O	0.17	0.09	0.16	0.11	0.20	0.21	0.22	0.21
SrO	0.14	0.19	0.15	0.12	0.21	0.15	0.16	0.15
Total	97.84	97.69	100.08	99.70	98.60	98.87	99.35	98.91
An	64.64	71.98	70.93	79.43	62.53	62.55	61.61	61.11

‘Core’ compositions are sampled close to melt inclusion

### Melt inclusion compositions

**Fig. 7:**
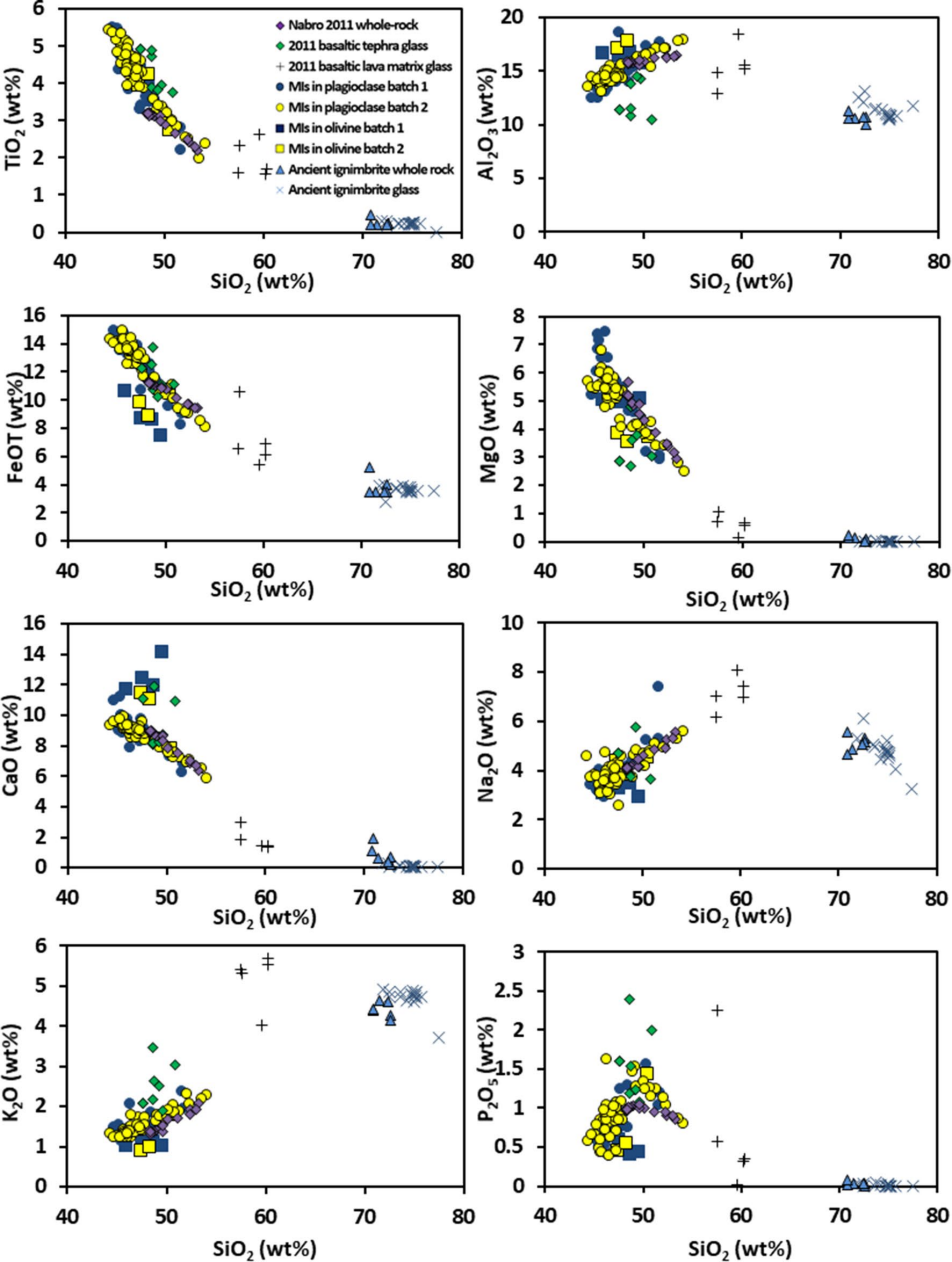
Major element Harker diagrams for whole rocks (purple diamonds), tephra glasses (green diamonds), lava glasses (black crosses), and melt inclusions in plagioclase (circles) and olivine (squares). Blue denotes samples from Batch 1; yellow is Batch 2. Also shown are ancient ignimbrite whole rock (light blue triangles) and ancient ignimbrite glass (light blue crosses). Whole-rock Fe_2_O_3_ recalculated as FeOT. All data normalised to 100% anhydrous

Figure 7 shows the melt inclusions, glass, and whole-rock compositions from the 2011 eruption, and whole-rock and glass compositions from the ancient ignimbrites for reference. As shown in Fig. 7, the 2011 magma is fractionated (Mg# ~ 40) and rich in TiO_2_ (~ 3 wt%) and total FeO (~ 12 wt%). More evolved samples are hypersthene normative, while those with < 50 wt% SiO_2_ are slightly nepheline normative. Melt inclusions are mostly nepheline normative regardless of SiO_2_ content. Melt inclusions are generally basaltic, trending to trachybasalt (Fig. 3) and follow the same evolutionary trend apparent in whole-rock compositions for older products of Nabro. However, an alkali-enriched trend is also present in some of the inclusions and in the matrix glass (Tables 4, 5), consistent with a greater role for clinopyroxene crystallisation in groundmass composition relative to phenocryst proportions. This suggests that the chemical processes responsible for generation of the groundmass glass chemistry (i.e., crystallisation at low pressure during eruption and ascent) differ from those responsible for the whole-rock trends.

**Table 4:** Representative glass analyses, measured using electron probe microanalysis

	NAB-11-4	NAB-11-78	NAB-11-53	NAB-11-12
	Lava	Lava	Tephra	Tephra
SiO_2_	58.6371	61.0837	48.7402	49.6084
TiO_2_	2.5785	1.7254	4.7249	3.9415
Al_2_O_3_	18.1245	15.345	10.7693	14.2698
FeOT	5.3103	7.0254	13.8071	11.0998
MnO	0.1067	0.1111	0.5189	0.2441
MgO	0.135	0.6591	2.6741	4.4892
CaO	1.4267	1.3847	8.123	8.7172
Na_2_O	7.9416	7.0637	4.1329	4.1254
K_2_O	3.9483	5.5937	3.4701	1.9028
P_2_O_5_	0.0133	0.361	2.4016	1.0809
SO_2_	0.0097	0.1136	0.0278	0.0285
Cl	0.0175	0.3994	0.2541	0.084
F	0	0.3257	0.5095	0.28
Total	98.3752	101.3474	100.1536	99.8941

**Table 5:** Representative melt inclusion compositions

	NAB11-S4-2-1	NAB11-M4-4-1	NAB11-M4-11-1	NAB11-S4-16-2	NAB11-M4-6-2	NAB11-S4-11-1	NAB11-M4-22-1	NAB11-M4-18-2	NAB11-M1-3-1	NAB11-S4-19-1
Sample	87	87	87	83	87	83	86	86	60	83
Batch	1	1	1	2	1	2	2	2	2	2
SiO_2_	48.63	47.49	46.41	46.82	48.20	45.62	54.16	46.98	53.62	51.37
TiO_2_	3.54	4.36	3.84	4.36	3.64	4.86	2.36	4.03	1.97	2.83
Al_2_O_3_	16.75	16.46	14.53	13.92	15.04	14.40	17.84	14.25	17.77	17.08
FeOT	8.65	8.77	13.53	13.79	12.33	14.04	8.06	13.46	8.49	9.32
MnO	0.05	0.10	0.27	0.18	0.22	0.14	0.24	0.20	0.19	0.16
MgO	4.88	4.98	6.53	5.35	5.02	5.53	2.48	5.56	2.78	3.39
CaO	12.00	12.43	9.27	9.14	8.60	9.34	5.82	8.49	6.45	7.16
Na_2_O	3.48	3.29	3.18	3.47	4.06	3.38	5.57	4.13	5.26	5.05
K_2_O	1.14	1.12	1.35	1.35	1.49	1.27	2.27	1.48	2.17	1.88
P_2_O_5_	0.41	0.60	0.40	0.93	0.76	0.48	0.80	0.93	0.86	1.24
Cr_2_O_3_	0.00	0.00	0.03	0.06	0.05	0.08	0.00	0.00	0.00	0.00
SO_2_	0.19	0.17	0.40	0.43	0.37	0.57	0.12	0.26	0.13	0.15
Cl	0.06	0.07	0.09	0.05	0.06	0.06	0.13	0.06	0.12	0.13
F	0.21	0.17	0.18	0.16	0.15	0.22	0.15	0.16	0.19	0.25
Total	100.00	100.00	100.00	100.00	100.00	100.00	100.00	100.00	100.00	100.00
H_2_O	1.32	1.34	1.23	1.52	1.44	0.54	2.10	1.08	1.91	1.59
H_2_O err	0.07	0.08	0.06	0.07	0.04	0.03	0.09	0.05	0.06	0.08
CO_2_	2160.81	2161.88	3455.99	774.45	1744.67	1138.85	202.57	1756.96	686.07	682.29
CO_2_ err	44.61	39.45	59.53	19.20	25.48	42.63	11.19	29.61	30.38	26.75
Sc	35.78	37.82	36.50	30.48	21.09	15.05	14.13	31.95	12.40	18.62
V	362.12	414.95	383.13		251.61	276.75	151.49	242.14		162.25
Sr	500.03	677.65	688.05		750.60	712.46	741.84	682.94		834.24
Y	24.23	31.68	32.80		35.84	23.01	40.60	37.68		44.85
Zr	221.37	243.00	244.34		257.25	173.52	336.11	278.15		303.44
Nb	53.46	57.72	57.07		59.99	41.91	80.38	68.35		77.50
Ba	365.67	334.03	403.27		456.08	302.27	701.17	477.69		564.71
La	29.55	39.44	42.86		49.82	31.45	61.76	56.90		62.95
Ce	63.82	83.14	92.98		104.93	64.27	129.33	116.51		134.60
Pr	7.98	10.18	11.00		12.63	7.71	14.65	14.20		16.36
Nd	30.22	40.66	44.47		50.19	31.97	57.72	55.47		64.91
Sm	6.10	8.60	7.82		10.78	7.14	10.52	11.35		13.79
Eu	1.98	2.52	3.14		3.54	2.00	4.60	3.44		4.44
Gd	0.52	7.16	9.17		10.53	6.11	12.33	9.19		14.32
Tb	0.74	1.13	1.37		1.51	0.89	1.69	1.81		1.95
Dy	5.29	4.73	6.02		7.84	4.11	7.60	7.11		8.25
Ho	0.94	1.16	1.20		1.38	0.78	1.63	1.40		1.64
Er	2.65	2.79	3.13		3.70	1.96	3.32	3.44		3.94
Tm	0.33	0.40	0.51		0.55	0.36	0.46	0.48		0.64
Yb	2.76	2.78	2.77		2.48	1.68	3.05	3.00		3.05
Lu	0.32	0.43	0.49		0.33	0.20	0.45	0.35		0.41
Hf	4.88	5.42	4.87		5.37	3.58	7.18	5.69		6.50
Ta	3.94	4.25	3.31		4.64	2.71	5.37	4.42		5.74
Th	3.31	4.13	3.56		4.16	2.87	7.54	4.58		6.36
U	1.09	0.92	1.00		1.23	0.77	1.89	1.15		1.52

All major elements measured by electron microprobe; trace elements, H_2_O and CO_2_ by ion probe. Errors quoted for ion-probe data are fully propagated Gaussian errors. Additional melt inclusion data are given in Supplementary Data

Major element compositions of the whole rocks reflect the phase assemblage—olivine, plagioclase, clinopyroxene, magnetite, ilmenite, and minor apatite. There is a clear peak in the SiO_2_–P_2_O_5_ plot at the point of apatite saturation. There is no TiO_2_ fractionation peak, suggesting that ilmenite was forming throughout crystallisation, though the slightly higher TiO_2_ in the glasses might indicate that magnetite (containing 17–24 wt% TiO_2_) dominated in the shallow system; this would be consistent with the magnetite mantles on chromite and its dominance in the groundmass. The differentiation trend in the whole rocks is less extensive than in the glasses. Some of the melt inclusions are more evolved than the groundmass, which may imply that these inclusions are contained in crystals that were stored in a crystal-rich magma reservoir before being remobilised prior to eruption. We show below that post-entrapment crystallisation of the host mineral cannot account for this behaviour.

Trace element contents are suggestive of a simple fractionation trend, though there is some scatter. Incompatible trace elements generally increase with decreasing MgO, while compatible elements decrease. Rare-earth elements (REEs) are generally enriched, and La/Yb in the whole rock is ~ 17.7–18.1 (PM-normalised 12.3–13.2).

The melt inclusions display considerable variation in their major element chemistry (Fig. 7)—generally much wider than that seen in the whole-rock data, though they follow it closely. The range of Mg# (54–35) is also greater, but MgO does not show a simple, negative linear correlation with incompatible elements such as La and Zr. Melt inclusions in olivine have consistently lower contents of FeO, K_2_O, and P_2_O_5_, and higher abundances of CaO and Al_2_O_3_ than those in plagioclase. They also have slightly lower abundances of incompatible trace elements. The inclusions in plagioclase track the liquid line of descent of the whole rocks closely.

**Fig. 8:**
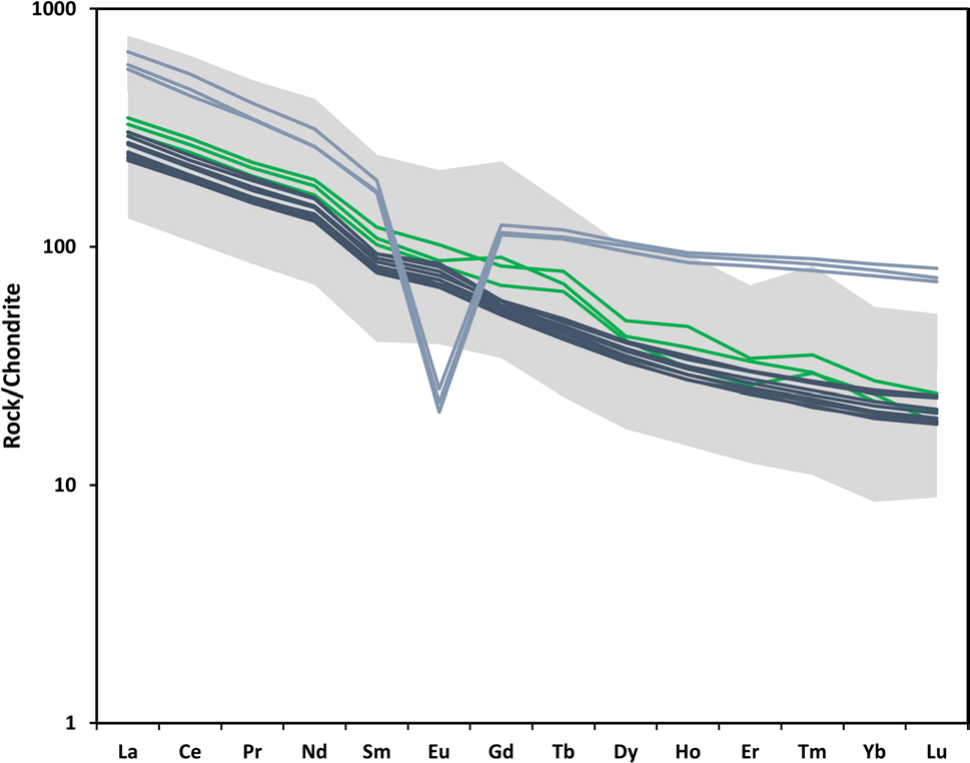
Rare-earth element patterns for the Nabro melt inclusions. Grey area denotes the range of compositions spanned by the melt inclusions. Dark blue lines represent the 2011 whole rock, green lines are matrix glasses, and light blue are the ignimbrite whole rock. The REEs have been normalised to chondrite (McDonough and Sun 1995)

Melt inclusions in plagioclase can be divided into two groups based on chemical zonation of the host phenocrysts (Fig. 6). The most evolved melt inclusions are found exclusively in unzoned phenocrysts or those with very slight reverse zoning. The most primitive inclusions are found in the high-An (75–80) cores of zoned phenocrysts, but there are some primitive inclusions in high-An unzoned phenocrysts. While both zoned and unzoned crystals are found in samples from both batches, the unzoned crystals are more common in Batch 1 (glassy). Melt inclusions in zoned plagioclase crystals are generally less evolved than those in unzoned crystals. All melt inclusions are strongly enriched in the LREEs relative to HREEs (Fig. 8).

#### Boundary-layer effects

A common cause of variation in melt inclusion composition is the influence of a boundary layer of melt around the crystal present during rapid growth (Baker 2008; Kent 2008; Métrich and Wallace 2008). This can affect slow-diffusing components, such as Al_2_O_3_ or P_2_O_5_, but would not impact Na_2_O or CaO due to their higher diffusivity. Furthermore, this effect is typically only found in small inclusions [< 15 µm; (Kuzmin and Sobolev 2003)], and is dominantly observed in experiments (e.g., Baker 2008). Since the beam size for the ion microprobe is > 30 µm, we did not study any inclusions smaller than this. Kent (2008) estimates that inclusions > 30 µm in diameter should be free from boundary-layer effects under natural magmatic conditions. The observed trends at Nabro for both melt inclusions and whole rocks show a clear fractionation peak for P_2_O_5_ (Fig. 7) that is hard to reconcile with boundary-layer effects.

#### Post-entrapment processes

##### Olivine

Post-entrapment crystallisation (PEC) for the olivine-hosted inclusions is < 4 wt%, based on an analysis using *Petrolog3* (Danyushevsky and Plechov 2011). Only three inclusions have lost FeOT relative to MgO. The calculated extent of PEC cannot explain the chemical differences between the plagioclase-hosted inclusions and those in olivine. In light of the limited evidence for PEC, we have not incorporated post-entrapment corrections to the data set, because such corrections would increase uncertainty rather than mitigate it—we present corrected compositions in supplementary data for reference.

##### Plagioclase

Post-entrapment diffusion of trace elements through plagioclase has been investigated by several authors (Cherniak 2003; Cottrell et al. 2002). Of particular interest are Sr, Ba, and Eu, which are compatible in plagioclase. We investigated this in comparison with the olivine-hosted melt inclusions and the whole-rock data. While the olivine-hosted inclusions are slightly lower in Sr, there are no significant differences between the two groups in Sr or Ba relative to either SiO_2_ or MgO, and no difference in the magnitude of the Eu anomaly (Eu/Eu* ~ 0.3). Again, we conclude that the difference in major element composition of the olivine-hosted inclusions and those in plagioclase cannot be explained by any post-entrapment or boundary-layer effects. The only major differences between the olivine-hosted inclusions and those in plagioclase are in Al_2_O_3_, FeOT, CaO, and K_2_O. This combination cannot be explained by post-entrapment processes, but could be explained by crystallisation order (“MELTS modelling”).

#### Presence of bubbles

We checked the melt inclusions with bubbles (present in < 20% of inclusions) but found no deviations from the trends observed in the full data set. We, therefore, do not believe that significant CO_2_ was present in the shrinkage bubbles.

### Melt inclusion volatile contents

**Fig. 9:**
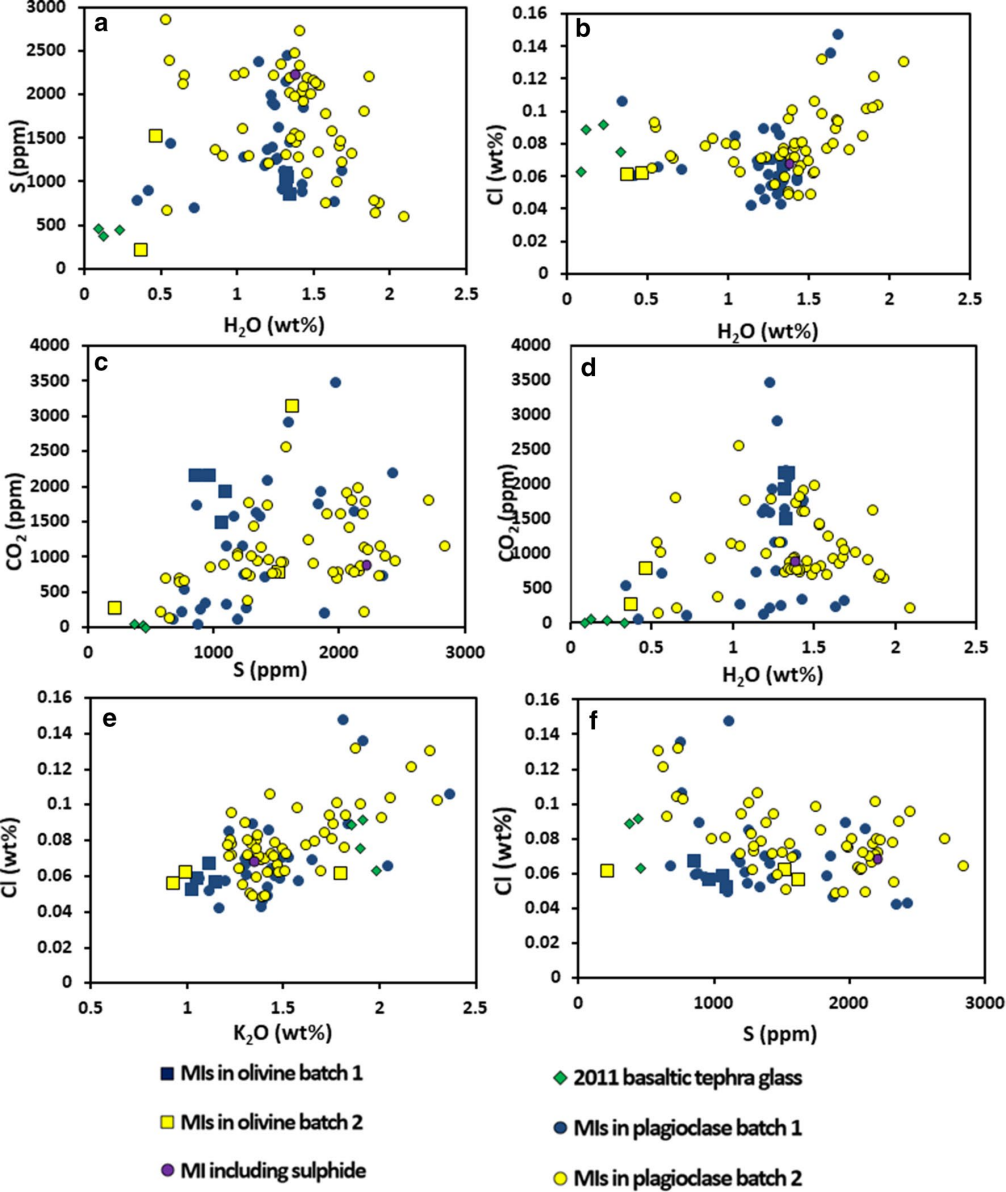
Melt inclusion and glass volatile contents

Melt inclusions contain up to 3455 ppm CO_2_, and 2.1 wt% H_2_O (Fig. 9). S/Cl in inclusions is relatively low (0.5–5 by mass); F/Cl is 0.5–5 by mass, with F up to 0.32 wt%. A few melt inclusions in xenocrysts have been measured by electron microprobe, but are rare. Two of these melt inclusions, one in plagioclase and one in olivine, contained > 7000 ppm F.

Sulphur contents range from 200 to 3000 ppm. They are more variable than Cl and F with which they show no correlation suggesting that these different volatile species degas at different pressures, consistent with experimental work (Lesne et al. 2011a). The peak in the S–H_2_O plot (Fig. 9a) at around 1 wt% H_2_O suggests that the partitioning of S into another phase (fluid or crystal) is enhanced at low pressures when the H_2_O content of the melt is low. The appearance of a small sulphide crystal in melt inclusions with the highest S contents (Fig. 9a) and the widespread presence of sulphide globules in the melt suggests that sulphide saturation plays a role in controlling the behaviour of sulphur. In contrast, the substantial decrease in S, at near-constant Cl (Fig. 9f), suggests that Cl becomes enriched by crystallisation, while S is actively partitioning into the fluid or sulphide phase, such that Cl degassing is a relatively late-stage process. The tendency of Cl to behave as an incompatible element is supported by its correlation with K_2_O (Fig. 9e). CO_2_ and S are weakly correlated (Fig. 9c). Although these components may partition similarly into the fluid phase, CO_2_ will not be sequestered by sulphide minerals and this may confer scatter. The CO_2_–H_2_O plot (Fig. 9d), like S–H_2_O, shows a maximum at intermediate H_2_O contents. It is clear that this cannot simply be a decompression degassing trend, as this would see a sharp drop in CO_2_ at near-constant H_2_O (Newman and Lowenstern 2002). Instead, the CO_2_–H_2_O behaviour is suggestive of other processes driving degassing as discussed below.

## Discussion

Here, we use the volatile and trace element data presented above to investigate the magmatic system feeding the 2011 eruption. Initially, we examine trends in volatile and trace element composition, and use this information to model intrinsic variables and draw inferences. We then examine the melt inclusion evidence for multiple batches of magma as identified from textures. Finally, we model the chemical data using rhyolite–MELTS (Gualda and Ghiorso 2015, 2012).

### Volatile-trace element systematics

**Fig. 10:**
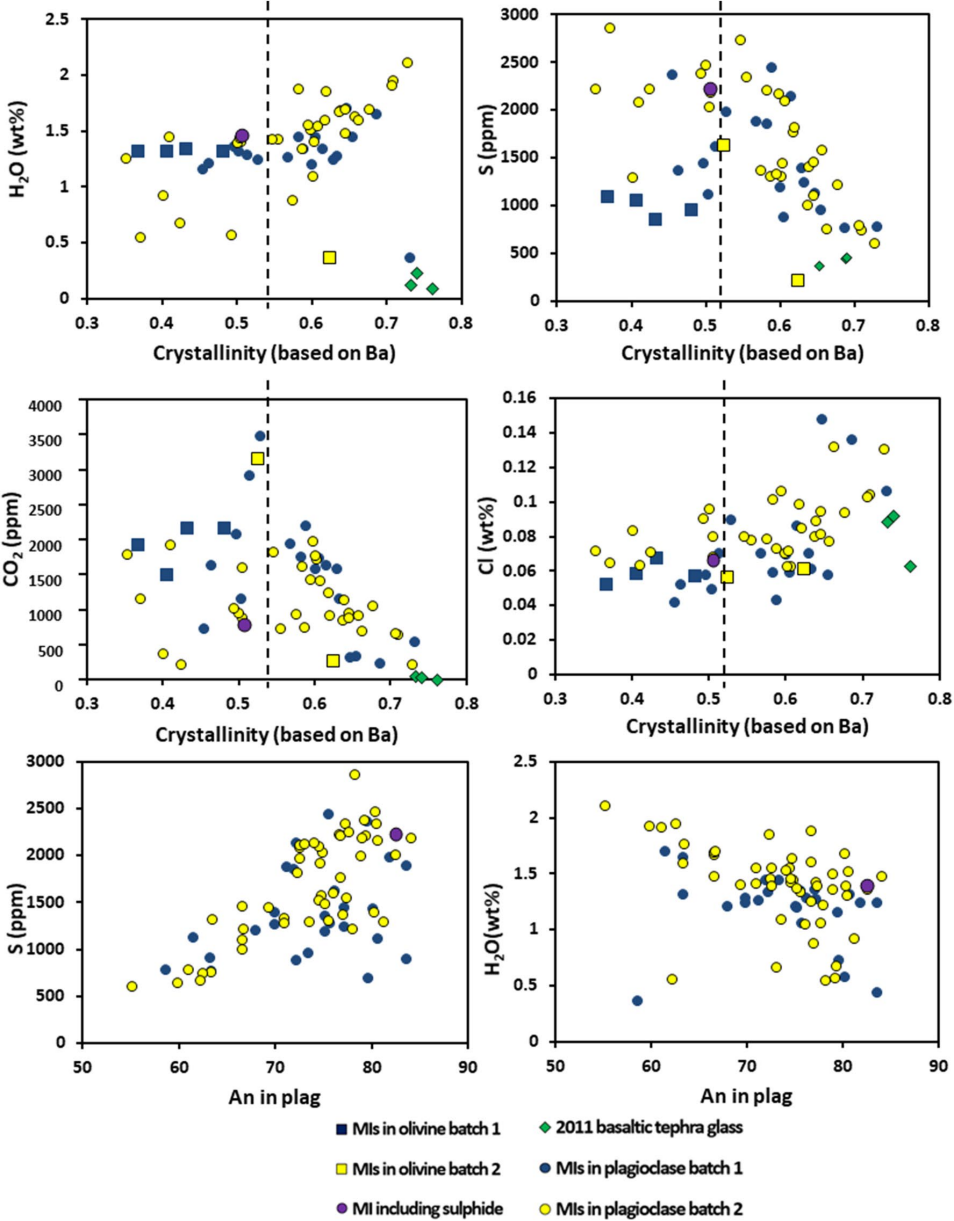
Melt inclusion and glass volatile contents plotted against crystallinity (calculated from Ba content, as described in text) and plagioclase anorthite content. The black dashed line marks the point of vapour saturation based on the CO_2_-crystallinity plot

Incompatible trace elements generally increase as volatile abundances decrease, except for the most primitive melt inclusions, which show a slight increase in CO_2_ with increasing incompatible trace elements. If we assume that the degree of melt crystallisation is equal to 1 − *F* (where *F* is melt fraction, *F* = *C*_l_/*C*_m_), and use a highly incompatible element (Ba) to calculate *F*, we obtain the relationship shown in Fig. 10, which shows that some of the melt inclusions increase in CO_2_ as crystallisation increases: there is a positive correlation between CO_2_ and incompatible trace elements (Ba, Th, and Zr). We suggest that these melt inclusions are vapour-undersaturated (Fig. 10), and, therefore, not suitable for the calculation of saturation pressures. However, we note that the high values of incompatible elements (particularly Nb and Ba), and correspondingly low values of CO_2_/Nb and CO_2_/Ba, are at odds with studies on other volatile-undersaturated basaltic samples (Hudgins et al. 2015; Le Voyer et al. 2017; Longpré et al. 2017; Rosenthal et al. 2015). We make two comments about this. First, very little is known about the mantle source composition beneath Afar, particularly for the marginal ranges, in terms of volatiles and incompatible trace elements. Thus, caution should be exercised when making comparisons to other volcanic settings. Second, if magmas have initially low volatile contents and undergo some crystallisation and melt inclusion entrapment at depth, then such melts will be vapour-undersaturated.

Four of the five olivine-hosted inclusions that are volatile-undersaturated have Fo_80_–Fo_88_, 1.3 wt% water and ~ 1500–2160 ppm CO_2_, whereas the volatile-saturated inclusion has Fo_71_, ~ 0.37 wt% H_2_O and 271 ppm CO_2_. Thus, volatile saturation only appears to have occurred following a degree of crystallisation, reducing olivine Fo content. This observation is consistent with relatively low initial volatile contents and some deep crystallisation and melt inclusion entrapment. Plagioclase anorthite content correlates positively with S and broadly negatively with both H_2_O and Cl; although there is a group of inclusions with high An and low sulphur, these are dominantly from Batch 1 (An 70–80; S 500–1000 ppm). These trends might suggest that for most inclusions, appreciable SO_2_ degassing preceded the degassing of water and Cl, consistent with relatively low initial H_2_O/S ratio. However, early sulphide saturation may also play a role. We note that the inclusion containing a sulphide globule is hosted in a phenocryst core An ~ 83, in a Batch 2 sample.

### Thermometry and barometry from minerals and melt inclusions

As shown in Fig. 7, there is a clear fractionation peak for P_2_O_5_ in our melt inclusion and whole-rock data. We have, therefore, applied an apatite saturation model (Harrison and Watson 1984; Pichavant et al. 1992) to estimate magma temperature for suitable inclusions. We also calculated pressure and temperature from the main mineral phases present using a variety of thermometers and barometers (Table 6). The models give temperatures ranging from 980 to 1146°C (with two outliers), but most inclusions indicate temperatures around 1080°C with *f*O_2_ between the QFM and NNO buffers. A touching pair of ilmenite and magnetite phenocrysts places *f*O_2_ of NNO + 0.04 (Ghiorso and Evans 2008) or NNO − 0.33 (Andersen and Lindsley 1988). Agreement between different thermometers is generally good (Table 6), with overlap between the melt inclusions and matrix glasses (no statistically significant differences if the clinopyroxene-melt thermometer is excluded).

**Table 6:** Intrinsic variables calculated using a range of models (Andersen and Lindsley 1988; Beattie 1993; Ghiorso and Evans 2008; Harrison and Watson 1984; Papale et al. 2006; Putirka 2005; Putirka et al. 2003, 2007; Sisson and Grove 1993; Spencer and Lindsley 1981)

Mineral	References	*T* (°C)	*P* (kbar)	*f*O_2_
Apatite	Harrison and Watson (1984)	980–1020		
Olivine–melt (incl)	Beattie (1993)	1066–1120		
	Putirka et al. (2007)	1037–1084		
	Sisson and Grove (1993)	992–1051		
Olivine–melt (matrix)	Beattie (1993)	1120–1146		
	Putirka et al. (2007)	1091–1126		
	Sisson and Grove (1993)	1029–1059		
Plagioclase–melt (inclusions)	Putirka (2005)	1063–1138		
Plagioclase–melt (matrix)	Putirka (2005)	1098–1108		
Ilmenite–magnetite	Spencer and Lindsley (1981)	1107		NNO − 0.04
	Andersen and Lindsley (1988)	1002		NNO − 0.33
	Ghiorso and Evans, (2008)	1013		NNO + 0.04
Cpx-melt	Putirka et al. (2003)	998–1104	2.3–5.2	
Melt inclusion (volatile-saturated)	Papale et al. (2006) Ghiorso and Gualda (2015)		0.2–4.6 0.2–2.6	

Oxide formulae recalculated according to Stormer (1983). Ilmenite–magnetite was calculated for a single touching pair. Crystal compositions were obtained either at the rim (for use with matrix compositions) or within 50 microns of the inclusion (melt inclusions)

Melt inclusions that are demonstrably vapour-saturated allow the calculation of saturation pressures. Mineral saturation temperatures can be obtained from the chemistry of the coexisting melt inclusion and its host mineral, provided that subsequent chemical diffusion has not modified their chemistry. Using the temperatures modelled for each inclusion following Putirka (2005) and the H_2_O–CO_2_ model of Papale et al. (2006), we obtain saturation pressures for those melt inclusions considered to be vapour saturated, from 50 to 500 MPa. These pressures correlate with those obtained from the *VolatileCalc* model of Newman and Lowenstern (2002), but are mostly higher. The solubility model of Ghiorso and Gualda (2015) yields similar pressures to those of *VolatileCalc*, and we have chosen to use these values in the following discussion. We infer that volatile-undersaturated melt inclusions were trapped at pressures higher than ~ 500 MPa (a full comparison of pressure and temperature models is provided in Supplementary Data).

**Fig. 11:**
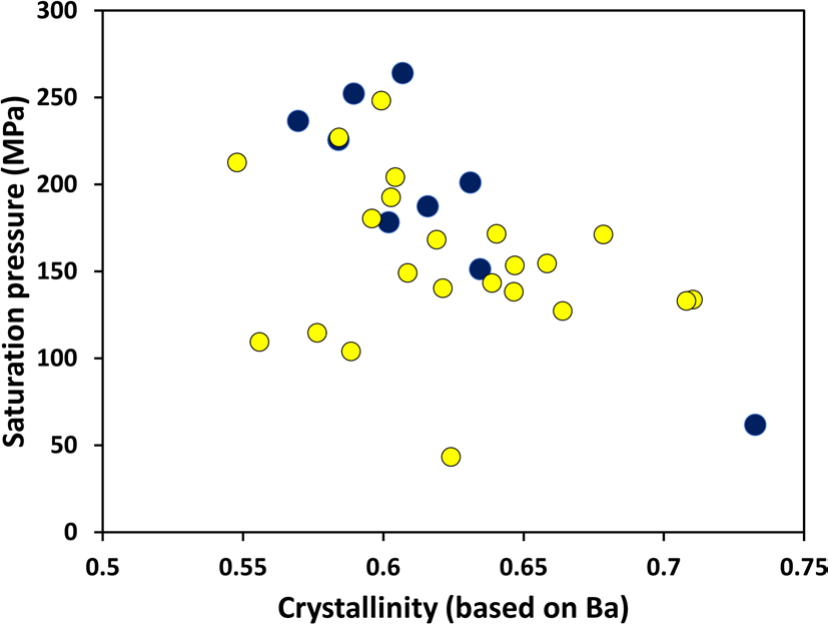
Crystallinity (calculated from Ba as before) and volatile saturation pressure (from Ghiorso and Gualda 2015) for melt inclusions, whose chemistry suggests that they were vapour-saturated at the time of entrapment

In Fig. 11, we plot pressure, calculated from solubility models, against melt fraction for the vapour-saturated inclusions. This demonstrates that the inclusions have not undergone simple isobaric crystallisation, in which case crystallinity would increase at near-constant pressure. Crystallisation does tend to increase with decreasing pressure, although the data do not describe a simple decompression crystallisation trend, as there is some scatter at any given pressure, notably at 200 MPa. In the next sections, we first return to the textural and chemical variations within the magmas, to explore the possibility that there are different evolutionary histories represented in the inclusions, and then model two potential processes that might explain the data.

### Evidence for multiple magma batches

#### Mineral evidence

Apatite saturation occurred at lower temperatures than are given by some of the melt inclusions, and probably occurred at shallow levels. There are two populations of apatite phenocrysts—one that contains appreciable sulphur (up to 500 ppm) and one that does not. Phenocrysts show some zoning in sulphur, where it is present, with rims being richer in sulphur than cores. Sulphur uptake by apatite is a function of *f*O_2_ as well as melt composition, and requires relatively oxidising conditions assuming that all S are incorporated as sulphate rather than sulphide (Boyce and Hervig 2008, 2009; Parat et al. 2008).

If we separate the two batches of magma based on our textural types, and plot the crystallinity of the melt against P_2_O_5_, we see that both batches were apatite-saturated at a late stage in their evolution, but large apatite phenocrysts are only found in Batch 2. This is further evidence of decompression crystallisation dominating the pre-eruption behaviour, as this would only increase temperature slightly due to latent heat release (Blundy et al. 2006).

As noted in “Petrography and mineralogy of 2011 samples”, there are a wide range of phenocryst compositions, sizes, and zoning patterns in the samples. There are also xenocrysts of all the major phases, plus anorthoclase and orthopyroxene (both of which exhibit disequilibrium textures). While large, zoned phenocrysts are mainly found in Batch 2 of the samples, there are a few in Batch 1, which might suggest some mixing between the batches—consistent with the textural evidence in Fig. 4, and with the observation that both batches contain phenocrysts with apatite-saturated inclusions.

#### Evidence from melt inclusion compositions

Using the model of Liu et al. (2007), we calculated the sulphur content at sulphide saturation for our melt inclusions. According to this model, almost all the inclusions should be sulphide-saturated. There are also sulphide globules in the groundmass of some of the samples. We identify two modal populations of sulphur content in our inclusions. The Kruskal–Wallis test suggests that the two groups can be ascribed to different samples. As some of the samples were obtained from a section through the 2011 tephra deposit, it seems likely that the groups represent separate, but co-erupted, magma batches with different sulphur contents; plausibly a sulphide phase had separated from one and not the other in response to small variations in *f*O_2_. A sulphide phase (pyrrhotite) is present in a single melt inclusion in plagioclase. Melt inclusions analysed for major elements only in a single unzoned clinopyroxene phenocryst contain up to 3100 ppm S.

**Fig. 12:**
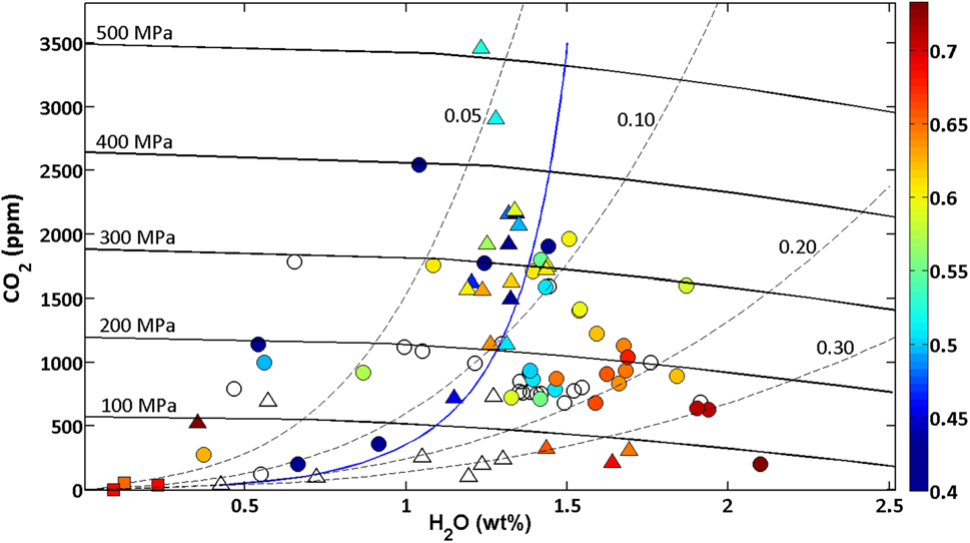
Model evidence for isothermal degassing and isobaric crystallisation. Filled symbols are melt inclusions for which trace element data allowed calculation of crystal fraction (colour scale, based on Ba); open black symbols are other melt inclusions. Triangles are Batch 1; circles are Batch 2. Dashed black lines are vapour isopleths in mole fraction H_2_O; and solid black lines are isobars (both isobars and isopleths calculated using VolatileCalc at 1080 °C). The blue line is an isothermal degassing model from VolatileCalc at 1.5 wt% H_2_O, 1080 °C

Statistical analysis of the melt inclusion data by sample further suggests that there are different batches of magma involved in the eruption. The Kruskal–Wallis test successfully distinguishes between samples that exhibit crystalline groundmass (Batch 2), and those that are glassy (Batch 1). We, therefore, classified melt inclusions according to Batches 1 and 2 and used the Kolmogorov–Smirnov test to investigate the data. This showed that inclusions in Batch 1 (glassy) have lower H_2_O and S and higher Mg# than those in Batch 2. However, both magmas appear to have reached apatite saturation. While there is considerable chemical overlap between the two batches, there are indications that Batch 2 underwent isobaric crystallisation in a storage region at ~ 200 MPa, where it increased in water content and became saturated in S (note a few inclusions that are increasing in crystallinity at constant pressure in Fig. 11, and a larger number in Fig. 12), while Batch 1 ascended from depth and stalled only briefly in the shallow crust.

### Isothermal degassing vs. isobaric crystallisation

Figure 12 shows the H_2_O vs. CO_2_ contents of the melt inclusions, along with isobars from VolatileCalc distinguished according to their crystallinity as calculated from Ba. One melt inclusion in olivine with high CO_2_ (3150 ppm) and very low water (0.2 wt%) is inferred to have lost water following entrapment (Bucholz et al. 2013). Melt inclusion volatile contents span a range of pressures (100–500 MPa) and vapour compositions. As noted above, the spread of the CO_2_–H_2_O data is not consistent with a simple closed or open degassing scenario; however, the data do offer insights into the sub-volcanic plumbing system, which evidently is vertically extensive. The data appear to define a broadly triangular region reminiscent of that described by Blundy and Cashman (2008, Fig. 31) for the case of a vertically-elongated plumbing system in which degassing and cooling occur concomitantly over a range of depths. The Blundy and Cashman (2008) model was developed for rhyolitic systems. We, therefore, derived a similar model, modified for basaltic melts (Fig. 12) to include both (1) isothermal degassing and (2) isobaric crystallisation. For (1), we assumed that degassing accompanied crystallisation during ascent, but that temperature remained constant at 1080 °C (i.e., we ignore contributions from latent heat release). For (2), we assumed that the magma was stored at a pressure of 200 MPa (~ 7 km depth) and that it was cooling and crystallising isobarically. The compositional dependence of volatile solubility in basalts introduces additional uncertainty into the model if we use the composition-sensitive model of Papale et al. (2006). Instead, for illustrative purposes, we used VolatileCalc for this, inputting the SiO_2_ for each inclusion separately and using average values for models.

The models in Fig. 12 suggest that Batch 1 of the magma ascended rapidly from depth and only degassed water at very shallow pressures (< 100 MPa), while Batch 2 (including the highly crystalline samples, red in Fig. 12) spent time in the shallow system (~ 200 MPa), increasing in H_2_O content due to isobaric crystallisation. This is supported by the evidence for apatite saturation and increasing water content at low CO_2_. There is evidently some crystal exchange between the batches, presumably due to crystal entrainment as one magma batch mingles with another during or shortly prior to eruption. The two textural batches are not clearly separated on any of the chemical plots that we present, and clearly have very similar sources. The evidence discussed earlier on relationships between S, An, and crystallinity suggests that a sulphide phase had separated from Batch 2, but possibly not from Batch 1. The mismatch between texture and chemistry supports the idea, proposed by Annen et al. (2006), that chemistry is a magmatic character derived in the melt source region at depth, whereas texture is a character determined by the magma ascent path and eruption dynamics. This is also backed up by the absence of apatite in Batch 1. It is likely that the two batches with different ascent and storage histories mixed together shortly before or during eruption. Mixing of magmas with similar chemistry but different textures has been described previously from the 2012–13 eruption of Tolbachik, Kamchatka (Plechov et al. 2015). We prefer this explanation rather than one involving clast recycling at the vent because of the differences in modal mineralogy of the samples, the presence of inclusions that are more evolved than groundmass, and the absence of apatite in Batch 1.

### MELTS modelling

**Fig. 13:**
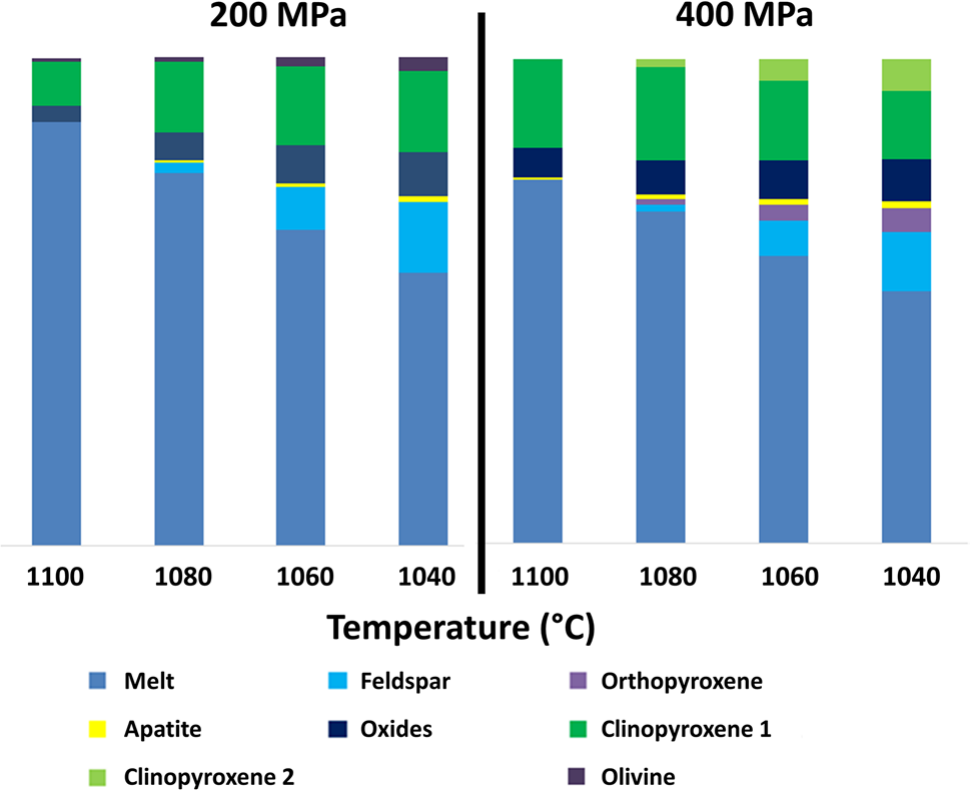
Rhyolite–MELTS results for isobaric crystallisation of trachybasalt (Sample 61) at **a** 2 MPa and **b** 400 MPa, at QFM and with 1.29 wt% H_2_O dissolved in the melt

We used the melt inclusion data above to perform MELTS modelling (Gualda et al. 2012), allowing us to integrate information from both the mineralogy and the melt inclusions. We modelled three separate processes, using a representative whole-rock composition (Sample 61: a trachybasaltic lava bomb from Batch 2—we also carried out models with Sample 12 from Batch 1, but the differences were minor) and 1.29 wt% dissolved H_2_O (consistent with the most primitive vapour-saturated inclusion). First, we modelled simple isobaric crystallisation at 400 MPa (Fig. 13). The model confirms that clinopyroxene rather than olivine is the liquidus phase, with oxides appearing rapidly thereafter and plagioclase at ~ 1088 °C. Olivine does not crystallise in this model, but apatite is stable at the lower temperature end. Orthopyroxene crystallises, although it is not observed in the natural samples with the exception of some rare relict xenocrysts. Second, we modelled isobaric crystallisation at 200 MPa. In this case, olivine is the liquidus phase, followed by clinopyroxene and spinel, with plagioclase appearing at 1085 °C. Interestingly, the phase assemblage at lower pressures is strongly influenced by *f*O_2_: at QFM, olivine precedes plagioclase at pressures lower than 175 MPa, but at NNO, plagioclase always crystallises first. Orthopyroxene is stable only at high pressures (e.g., 400 MPa) and low temperatures, suggesting that the xenocrysts in the natural samples (see “Melt inclusion compositions”) may be derived from a deep crustal reservoir.

Decompression crystallisation models similarly show clinopyroxene on the liquidus, with plagioclase and then olivine appearing at lower temperature. We tried a series of models from 600 to 100 MPa, some isothermal and others with decreasing temperature. In all of these models, H_2_O contents did not attain saturation levels (but the models ignore CO_2_, which would lead to earlier saturation). When pressure drops below 40 MPa, water degasses. In isothermal models, plagioclase is stable at 600 MPa below 1090 °C, which is consistent with the high saturation pressures obtained from the inclusions. Orthopyroxene destabilises at shallow pressures and lower temperatures, consistent with the textural evidence. Additional MELTS results are shown in the Supplementary Data.

MELTS modelling in alkaline rocks is challenging, and each of the models that we ran offers some insights into the magmatic system without producing results that match fully the data. Model results consistently suggest that clinopyroxene is the liquidus phase, but the order and onset of plagioclase and olivine crystallisation vary considerably, with both possible at high pressures, consistent with the melt inclusions. In some models, olivine crystallises considerably earlier than plagioclase, which might explain the high CaO and Al_2_O_3_ in the olivine inclusions relative to the plagioclase ones. The challenges of modelling alkaline rocks are discussed by Gleeson et al. (2017) and Rooney et al. (2012a). Rooney et al. (2012a) particularly note the problem that MELTS is not well calibrated for fluorapatite, something also evident in our results. Overall, however, modelling suggests that the magmas experienced a combination of isobaric cooling and degassing during decompression.

### Magma storage depths and plumbing system at Nabro

Using a crustal density of 2780 kg m^−3^ (Makris and Ginzburg 1987), we calculate that most melt inclusions were trapped at 5–10 km depth (consistent with seismic data for the depth of a magma body; Hamlyn et al. 2014), but some may have been trapped close to the base of the crust, around 20 km depth (Hammond et al. 2011). The magma storage region is most likely composed of a series of sills and old eruptive products, evidenced by the presence of xenocrystic material in the magmas. The mineralogy, textures, and melt inclusion composition of the 2011 eruption suggests that, based on textures, at least two batches of magma with different ascent and storage characteristics, but similar bulk compositions, were involved in the eruption.

**Fig. 14:**
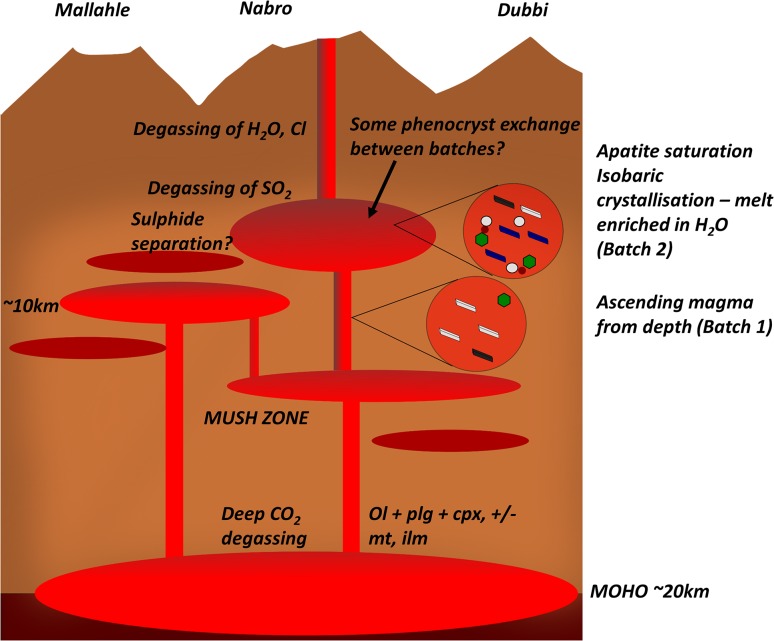
Schematic cross section showing the main findings of this study. Melt lenses are red; mush zone is light brown; and gas is shown as grey circles within the melt lenses

The major and trace element composition of the inclusions suggests that their source region is most likely the same as that of older rocks found at Nabro and Dubbi volcanoes—indeed, older and more primitive basalts from the Edd Volcanic Field (Dubbi) provide a plausible “parental” composition for this magma (De Fino et al. 1978; Figs. 3, 14).

Alkali basalts have been shown experimentally to have higher volatile solubilities than other compositions due to weaker polymerisation (Dixon 1997; Dixon et al. 1997; Iacovino et al. 2013; Lesne et al. 2011b, c; Vetere et al. 2014). The Nabro lavas are higher in alkalis than other volcanic products in the Afar, probably due to a smaller initial melt fraction in the mantle source region, consistent also with their enrichment in REE and in LREE/HREE. The high solubility of CO_2_ in alkaline magmas supports the finding that some of the more primitive inclusions are volatile-undersaturated and represent the original volatile contents of the magma, which is relatively high in water (~ 1.3 wt%) for an intraplate basaltic magma. The most primitive of these melt inclusions was trapped at the base of the crust, suggesting a deep origin for their plagioclase and olivine hosts. It is likely that the magma was crystallising both these phases, plus clinopyroxene (positive correlation between MgO and CaO/Al_2_O_3_), and an Fe–Ti oxide phase (evidenced from TiO_2_ fractionation) as it rose through the lower crust. At shallow depths, the magma became saturated in apatite, and a sulphide phase separated. Apatite saturation appears to have preceded most water degassing: phosphorus behaves incompatibly in most inclusions, but does drop in those that are most rich in water. Water degassing occurred at relatively low pressures, < 1 kbar, but there are very few melt inclusions trapped at such shallow depths. Sulphur, however, decreases consistently with decreasing depth, suggesting that some SO_2_ was degassing throughout magma ascent. The melt inclusions can be modelled by a combination of isobaric crystallisation and closed-system degassing during ascent. It is likely that they represent two or more batches of melt sourced from a single and relatively homogeneous melt zone in the uppermost mantle (~ 20 km depth; Hammond et al. 2011), inferred on the basis of mineralogy and sulphur content. Melts ascending through the crust may crystallise through decompression, once water saturation is attained, or stall and crystallise by cooling.

Other work in the wider East African Rift, examining the Main Ethiopian Rift and Kenyan Rift zones, has reached similar conclusions about crustal mush zones (e.g., Rooney et al. 2012a, b; Gleeson et al. 2017). These papers indicate that volcanic systems in the Main Ethiopian Rift are characterised by vertically extensive magma plumbing that are likely compositionally zoned. Field et al. (2012b), however, working on another Afar volcano, posited a system of multiple sills to explain their data. Nabro, similarly, appears to have distinct batches of magma probably stored in sills, within a mush zone that likely consists of a wide range of compositions (evidenced by xenoliths in the erupted products, as well as by the presence of a wide range of compositions in older rocks).

Combining our observations, we suggest that the crust beneath Nabro and the other Bidu volcanoes comprise a vertically extensive mush zone, within which sit multiple intrusions of melt that have risen from the base of the crust and are in various stages of crystallisation and fluid saturation (Fig. 14). In the 2011 eruption, an intrusion of fresh trachybasalt (Batch 1) rose through the crust, and encountered an older body of trachybasaltic melt (Batch 2), which was remobilised and erupted, with some mingling between batches. This would explain the occurrence of different textural batches with similar bulk chemistry. It is plausible that gravitational instability in the mush is important in triggering eruption as suggested for eruptions of andesite magmas at Soufrière Hills volcano, Montserrat (Christopher et al. 2015). Mush destabilisation can also lead to copious release of SO_2_ gas previously stored at different levels, accounting for the large emission of SO_2_ during the 2011 eruption of Nabro relative to the sulphur content that could have been contained within the erupted magma (Bourassa et al. 2012).

## Electronic supplementary material

Below is the link to the electronic supplementary material.

Supplementary material 1 (DOCX 446 KB)

Supplementary material 2 (XLSX 95 KB)

Supplementary material 3 (XLSX 19 KB)

Supplementary material 4 (XLSX 186 KB)

Supplementary material 5 (XLSX 39 KB)

## Footnotes

1 Mg# is the molar Mg/(Mg + Fe) ratio using total Fe.
