# Supplementary material for: The 2011 eruption of Nabro volcano, Eritrea: perspectives on magmatic processes from melt inclusions
Source: Contrib Mineral Petrol. 2017 Nov 27;173(1):1. doi: 10.1007/s00410-017-1425-2 (PMC6954031; doi:10.1007/s00410-017-1425-2)
Supplement: Supplementary file 1 — Supplementary material 1 (DOCX 446 KB) [file 410_2017_1425_MOESM1_ESM.docx]

# Supplementary data associated with “The 2011 eruption of Nabro volcano, Eritrea: perspectives on magmatic processes from melt inclusions”, Contributions to Mineralogy and Petrology

## Amy Donovan^1^, Jon Blundy^2^, Clive Oppenheimer^3^, Iris Buisman^4^

1 Department of Geography, King’s College London

Email: [amy.donovan@kcl.ac.uk](mailto:amy.donovan@kcl.ac.uk); Tel: 0207 848 1755

2 School of Earth Sciences, University of Bristol

3 Department of Geography, University of Cambridge

4 Department of Earth Sciences, University of Cambridge

This file contains (i) working curves for the ion probe data; (ii) a comparison of pressure models and thermometers; (iii) example MELTS results.

## Supplementary data: working curves

Water measurements

Day 1

Day 2

Day 3

Day 4

Standards used:

EIMF ST1, ST2, ST3, ST6

cgp1

pich2

jc4

BIR

Nist610

Bcr2

CO2 measurements

Standards: siss51, nist610, RB497, st1, st2, st6, 150-S, CPG1

Day 1

Day 2

Day 3

## Pressure model comparison

Squares – inclusions in olivine; circles – inclusions in plag

Filled– saturated inclusions; open – undersaturated (minima).


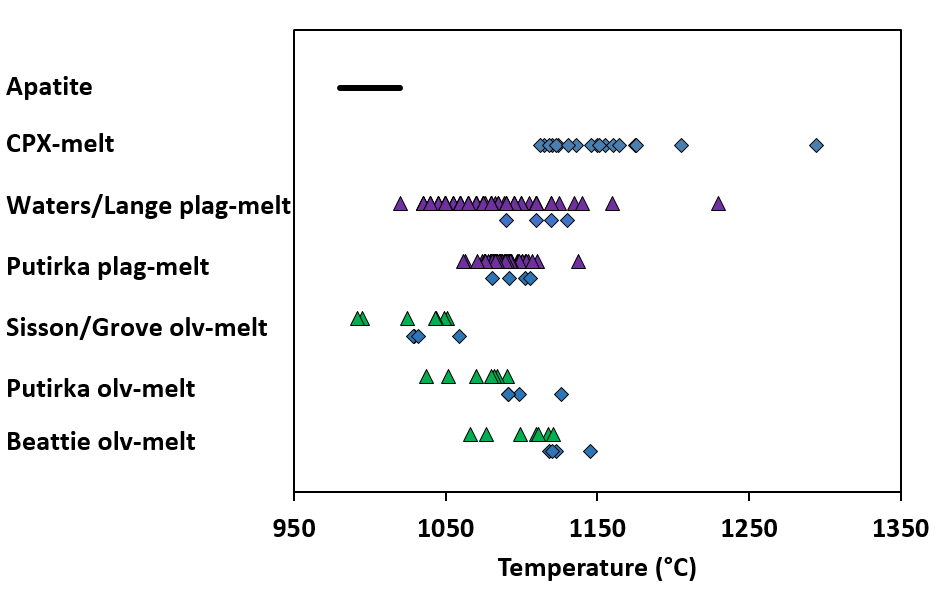


Temperature comparison – purple triangles = plag MI; green = olivine MI; blue diamonds = matrix glass; black line is the range of the apatites.

## Additional MELTS results


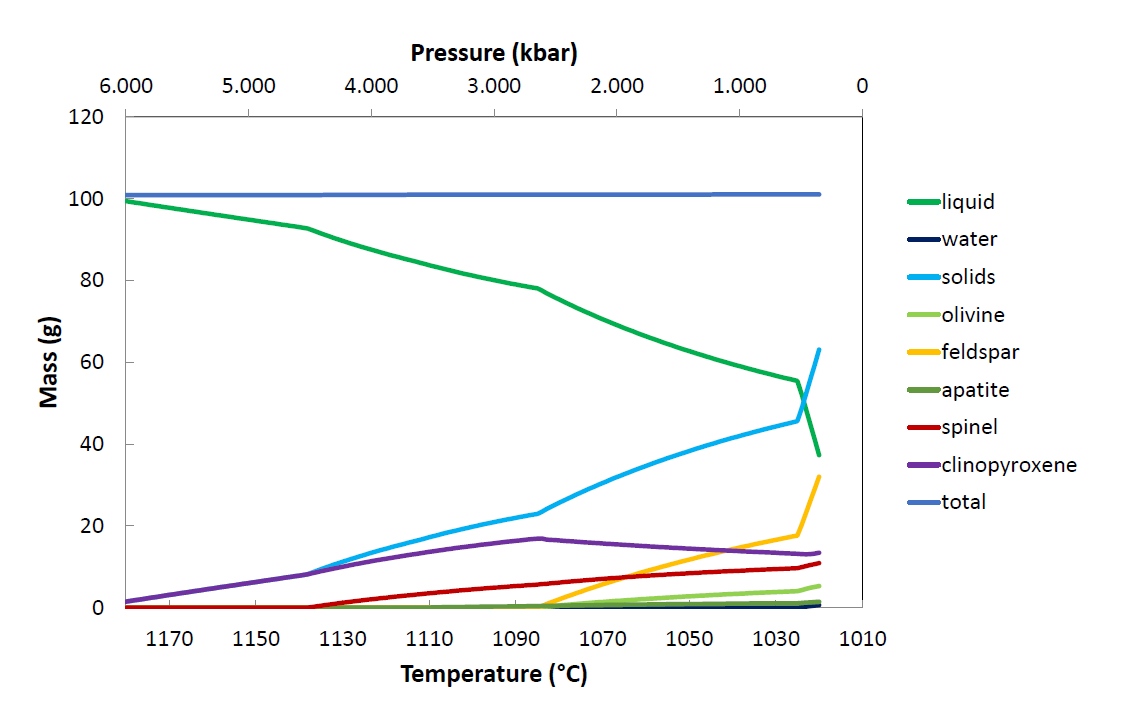


Example model for decompression crystallisation, using sample 61 WR at QFM. The following Harker diagrams show this model (filled blue diamonds) alongside others for isobaric crystallisation.
